# Supplementary material for: Protocol for a controlled human infection with genetically modified Neisseria lactamica expressing the meningococcal vaccine antigen NadA: a potent new technique for experimental medicine
Source: BMJ Open. 2019 May 1;9(4):e026544. doi: 10.1136/bmjopen-2018-026544 (PMC6501966; doi:10.1136/bmjopen-2018-026544)
Supplement: Supplementary data [file bmjopen-2018-026544supp001.pdf]

SUPPLEMENTARY TABLE 1: ELIGIBILITY CRITERIA

Inclusion criteria

Challenge volunteers	Contact volunteers
Healthy adults aged 18 to 45 years inclusive on the day of enrolment	Healthy adults aged 18 years or over on the day of enrolment
Fully conversant in the English language	
Able and willing (in the investigator’s opinion) to comply with all study requirements	
Provide written informed consent to participate in the trial	
Provide written agreement to abide by infection control guidelines including agreement to abstain from intimate contact with any individual other than one declared and consented bedroom contact during the study period	
Provide written consent to allow the study team to discuss the volunteer’s medical history with the General Practitioner	
Written informed contact volunteer consent provided by any bedroom contact	
Agreement to be admitted to Southampton NIHR-CRF for 4.5 days following inoculation	
For females only, willingness to practice continuous effective contraception (see below) during the study and a negative pregnancy test on the day(s) of screening and inoculation	For females only, willingness to practice continuous effective contraception (see below) during the study and a negative pregnancy test on the day of screening and challenge volunteer discharge
Agreement to take antibiotic eradication therapy according to the study protocol	
Able to correctly answer all questions in the pre-consent and infection control questionnaires	Able to correctly answer all questions in the infection control questionnaire
TOPS registration completed and no conflict found	

NIHR-CRF: National Health Institute for Health Research-Clinical Research Facility, TOPS: The Over-volunteering Prevention System

Effective contraception for female volunteers

Established use of oral, injected or implanted hormonal methods of contraception
Placement of an intrauterine device or intrauterine system
Total abdominal hysterectomy
Barrier methods of contraception (condom or occlusive cap with spermicide)
Male sterilisation if the vasectomised partner is the sole partner for the subject
True abstinence when this is in line with the preferred and usual lifestyle of the subject

Exclusion criteria

<table>
<tr><td>Challenge volunteers</td><td>Contact volunteers</td></tr>
<tr><td>Current active smokers defined as having smoked a cigarette or cigar in the last four weeks</td><td></td></tr>
<tr><td>N. lactamica or N. meningitidis detected on throat swab or nasal wash taken at screening or at the pre-challenge visit</td><td></td></tr>
<tr><td>Individuals who have a current infection at the time of inoculation</td><td></td></tr>
<tr><td colspan="2">Individuals who have been involved in other clinical trials involving receipt of an investigational product over the last 12 weeks or if there is planned use of an investigational product during the study period</td></tr>
<tr><td>Individuals who have previously been involved in clinical trials investigating meningococcal vaccines or experimental challenge with N. lactamica</td><td></td></tr>
<tr><td>Individuals who have received one or more doses of the meningococcus B vaccine Bexsero</td><td></td></tr>
<tr><td>Use of systemic antibiotics within the period 30 days prior to the challenge</td><td></td></tr>
<tr><td colspan="2">Any confirmed or suspected immunosuppressive or immune-deficient state, including HIV infection; malignancy, asplenia; recurrent, severe infections and chronic (more than 14 days) immunosuppressant medication within the past 6 months (topical steroids are allowed)</td></tr>
<tr><td>Use of immunoglobulins or blood products within 3 months prior to enrolment.</td><td></td></tr>
<tr><td>History of allergic disease or reactions likely to be exacerbated by any component of the inoculum</td><td></td></tr>
<tr><td colspan="2">Contraindications to the use of ciprofloxacin, specifically a history of epilepsy, prolonged QT interval, hypersensitivity to quinolones or a history of tendon disorders related to quinolone use</td></tr>
<tr><td colspan="2">Contraindications to the use of ceftriaxone, specifically hypersensitivity to any cephalosporins</td></tr>
<tr><td>Any clinically significant abnormal finding on clinical examination or screening investigations. In the event of abnormal test results, confirmatory repeat tests will be requested.</td><td></td></tr>
<tr><td colspan="2">Any other significant disease, disorder, or finding which may significantly increase the risk to the volunteer because of participation in the study, affect the ability of the volunteer to participate in the study or impair interpretation of the study data.</td></tr>
<tr><td colspan="2">Occupational, household or intimate contact with immunosuppressed persons, specifically HIV infection with a CD4 count <200 cells/mm3; asplenia; any malignancy, recurrent, severe infections and chronic (more than 14 days) immunosuppressant medication within the past 6 months (topical steroids are allowed)</td></tr>
<tr><td colspan="2">Occupational or household contact with children under 5 years or an older child with a tendency to co-sleep with the volunteer</td></tr>
<tr><td colspan="2">Pregnancy, lactation or intention to become pregnant during the study</td></tr>
<tr><td>Inability of the study team to contact the volunteer's GP to confirm medical history and safety to participate</td><td></td></tr>
</table>
