# Supplementary material for: Protocol for a controlled human infection with genetically modified Neisseria lactamica expressing the meningococcal vaccine antigen NadA: a potent new technique for experimental medicine
Source: BMJ Open. 2019 May 1;9(4):e026544. doi: 10.1136/bmjopen-2018-026544 (PMC6501966; doi:10.1136/bmjopen-2018-026544)
Supplement: Supplementary data [file bmjopen-2018-026544supp002.pdf]

SUPPLEMENTARY TABLE 2: INFECTION CONTROL GUIDELINES

During admission – challenge volunteers only:

- The volunteer must wear a surgical mask covering the nose and mouth at all times unless within their personal room, while showering or having respiratory samples taken or while outside in open air
- The volunteers are not allowed to enter the personal rooms of other volunteers
- The volunteer must wash his/her hands before leaving their personal room
- The volunteer is not allowed to leave the NIHR-CRF without permission of the clinical team
- Volunteers are allowed to leave the NIHR-CRF for a maximum of two hours twice a day, between 08.00-18.00
- The volunteer will be escorted by a member of the study team when walking through non-designated areas of the NIHR-CRF
- The volunteer must not have contact with immunosuppressed individuals
- The volunteer must not have any direct contact that could involve transfer of respiratory secretions to anyone during the admission period
- The volunteer must not use the main entrance of the hospital or shops or cafes within the hospital building
- When outside of the NIHR-CRF the volunteer must be contactable by mobile phone at all times and must have study emergency phone number stored on their phone to contact the clinical study team if necessary
- The volunteer must be able to be return to the NIHR-CRF within 30 minutes.
- The volunteer may receive a maximum of two guests at a time between 8.00 and 22.00, who must wear masks covering nose and mouth while in close proximity to the volunteer and must adhere to strict infection control procedures.

Following discharge – challenge and contact volunteers:

For the first two weeks following discharge volunteers must avoid crowded social environments such as pubs and clubs.

For the remainder of the study period:

- Volunteers must not have any contact with high risk of transmission with any individuals other than their declared and consented bedroom contact/corresponding challenge volunteer – such contact includes:
    - Bed sharing
    - Intimate/sexual contact
    - Contact that may involve transfer of respiratory secretions e.g. kissing
    - Sharing cutlery or drinking vessels
- Volunteers must not engage in oral sex
- Volunteers must avoid contact with immunosuppressed individuals
