# Supplementary material for: Protocol for a controlled human infection with genetically modified Neisseria lactamica expressing the meningococcal vaccine antigen NadA: a potent new technique for experimental medicine
Source: BMJ Open. 2019 May 1;9(4):e026544. doi: 10.1136/bmjopen-2018-026544 (PMC6501966; doi:10.1136/bmjopen-2018-026544)
Supplement: Supplementary data [file bmjopen-2018-026544supp003.pdf]

SUPPLEMENTARY TABLE 3 – STUDY TIMETABLE FOR CHALLENGE VOLUNTEERS

	Screening	Pre challenge	Admission					Follow up							Potential additional visits		
Timeline (days)	≤ 90	-5	0	1	2	3	4	7	10	14	28	56	90	92	Additional shedding check[b]	Triggered eradication[c]	Post triggered eradication check
Day		W	M	Tu	W	Th	F	M	Th	M	M	M					
Visit window		+/-2	0	0	0	0	0	+/-1	+/-1	+/-2	+/-3	+/-5	+/-7	-1 to 0[a]	0[d]	0[d]	0[a]
TOPS confirmation	+																
Volunteer Information Sheet	+																
Informed consent	+																
Infection control training	+						+										
Vital signs	+	(+)	+	+	+	+	+	(+)	(+)	(+)	(+)	(+)	(+)	(+)	(+)	(+)	(+)
Medical history	+																
Physical examination	+	(+)	(+)	(+)	(+)	(+)	(+)	(+)	(+)	(+)	(+)	(+)	(+)	(+)	(+)	(+)	(+)
Pregnancy test (females	+		+										+			+	
Urinalysis	+																
Electrocardiogram	+																
Review eligibility		+	+														
Inoculation			+														
Eradication													+			+	
Review of adverse events and concomitant medications		+	+	+	+	+	+	+	+	+	+	+	+	+	+	+	+
Throat swab 1	+	+	+		+	+	+	+	+	+	+	+	+	+	+	+	+
Nasal wash		+				+				+	+	+	+			+	
Throat swab 2 (microbiome)			+			+		+	+	+	+	+	+			+	
Nasosorption test			+			+				+	+	+	+			+	
Saliva sample			+			+				+	+	+	+			+	
Environmental samples				+	+	+	+	+	+	+	+	+	+		+	+	
Safety bloods	8		8			8		8		8	8	8	8			8	
Immunological blood tests			70					70		70	70	70	70			70	
Cumulative blood volume	8		86			94		172		250	328	406	484				

(+) If clinically indicated, [a]1-2 days after eradication, [b]If increased shedding seen at one timepoint from Day 14, [c]If early eradication triggered (see section 9.5.3), [d]As soon as possible after triggering results are known.
