# Supplementary material for: Protocol for a controlled human infection with genetically modified Neisseria lactamica expressing the meningococcal vaccine antigen NadA: a potent new technique for experimental medicine
Source: BMJ Open. 2019 May 1;9(4):e026544. doi: 10.1136/bmjopen-2018-026544 (PMC6501966; doi:10.1136/bmjopen-2018-026544)
Supplement: Supplementary data [file bmjopen-2018-026544supp004.pdf]

SUPPLEMENTARY TABLE 4 – STUDY TIMETABLE FOR CONTACT VOLUNTEERS

	**Screening**	**Challenge volunteer discharge**	**Follow up**					**Potential additional visits**	
Timeline (days)	≤ 90	4	14	28	56	90	92	Early / triggered eradication[c]	Early / triggered eradication check
Day		F	M	M	M	+/-7[a]	-1 to 0[c]	0[d]	-1 to 0[b]
Visit window		0	+/-2	+/-3	+/-5				
TOPS confirmation	+								
Volunteer Information Sheet	+								
Informed consent	+								
Reconfirm eligibility		+							
Infection control training	+	+							
Vital signs	+		(+)	(+)	(+)	(+)	(+)	(+)	(+)
Medical history	+								
Physical examination	+		(+)	(+)	(+)	(+)	(+)	(+)	(+)
Pregnancy test (females only)	+	+				+		+	
Urinalysis	+								
Electrocardiogram	+								
Eradication						+		+	
Review of adverse events and concomitant medications			+	+	+	+	+	+	+
Throat swab	+	+	+	+	+	+	+	+	+

(+) If clinically indicated, [a]Same day as corresponding challenge volunteer, [b]1-2 days after eradication, [c]If early eradication triggered, [d]As soon as possible after triggering results are known
