# Supplementary material for: Protocol for a controlled human infection with genetically modified Neisseria lactamica expressing the meningococcal vaccine antigen NadA: a potent new technique for experimental medicine
Source: BMJ Open. 2019 May 1;9(4):e026544. doi: 10.1136/bmjopen-2018-026544 (PMC6501966; doi:10.1136/bmjopen-2018-026544)
Supplement: Author's manuscript [file bmjopen-2018-026544.draft_revisions.pdf]

BMJ Open

BMJ Open is committed to open peer review. As part of this commitment we make the peer review history of every article we publish publicly available.

When an article is published we post the peer reviewers' comments and the authors' responses online. We also post the versions of the paper that were used during peer review. These are the versions that the peer review comments apply to.

The versions of the paper that follow are the versions that were submitted during the peer review process. They are not the versions of record or the final published versions. They should not be cited or distributed as the published version of this manuscript.

BMJ Open is an open access journal and the full, final, typeset and author-corrected version of record of the manuscript is available on our site with no access controls, subscription charges or pay-per-view fees (http://bmjopen.bmj.com).

If you have any questions on BMJ Open's open peer review process please email info.bmjopen@bmj.com

BMJ Open

Protocol for a controlled human infection with genetically modified Neisseria lactamica expressing the meningococcal vaccine antigen NadA: A potent new technique for experimental medicine

Journal:	*BMJ Open*
Manuscript ID	bmjopen-2018-026544
Article Type:	Protocol
Date Submitted by the Author:	12-Sep-2018

Complete List of Authors:	Gbesemete, Diane; University Hospital Southampton NHS Foundation Trust, NIHR Clinical Research Facility; University of Southampton , Faculty of Medicine<br>Laver, Jay; University of Southampton<br>de Graaf, Hans; University Hospital Southampton NHS Foundation Trust, NIHR Clinical Research Facility; University of Southampton , Faculty of Medicine<br>Ibrahim, Muktar; University of Southampton, Clinial & Experimental Sciences<br>Vaughan, Andrew; University of Southampton, Clinical & Experimental Sciences<br>Faust, Saul; University of Southampton, UK, NIHR Wellcome Trust Clincial Research Facility<br>Gorringe, Andrew; Public Health England Porton, Research<br>Read, Robert; University of Southampton; NIHR Southampton Biomedical Research Centre

Keywords:	INFECTIOUS DISEASES, meningitis, Neisseria meningitidis, genetically modified organisms, controlled human infection model

SCHOLARONE™ Manuscripts

Protocol for a controlled human infection with genetically modified *Neisseria lactamica* expressing the meningococcal vaccine antigen NadA: A potent new technique for experimental medicine

Authors:

Diane Gbesemete[1,2], Jay R Laver[3], Hans de Graaf[1,2], Muktar Ibrahim[1], Andrew Vaughan[2,3], Saul Faust[2,3], Andrew Gorringe[4], Robert C Read[1,3]

1. Faculty of Medicine, University Hospital Southampton NHS Foundation Trust, Southampton, UK
2. NIHR Clinical Research Facility, University Hospital Southampton NHS Foundation Trust, Southampton, UK
3. NIHR Southampton Biomedical Research Centre, University of Southampton
4. Pathogen Immunology Group, Public Health England, Salisbury, Wiltshire, UK

Corresponding author:

Diane Gbesemete, NIHR Clinical Research Facility, University Hospital Southampton NHS Foundation Trust, Southampton, SO30 3RE, UK

d.gbesemete@soton.ac.uk, 023 8120 4989

Key words:

Human challenge study, colonisation, *Neisseria lactamica*, Genetically modified organism

Word count:

5250

For peer review only

ABSTRACT

Introduction

Neisseria lactamica is a commensal organism found in the human nasopharynx and is closely related to the pathogen *Neisseria meningitidis* (meningococcus). Carriage of *N. lactamica* is associated with reduced meningococcal carriage and disease. We summarise an ethically approved protocol for an experimental human challenge study using a genetically modified strain of *N. lactamica* that expresses the meningococcal antigen NadA. We aim to develop a model to study the role of specific bacterial antigens in nasopharyngeal carriage and immunity, to evaluate vaccines for their efficacy in preventing colonisation, and to provide a proof of principle for the development of bacterial medicines.

Methods and analysis

Healthy adult volunteers aged 18-45 years will receive an intranasal inoculation of either the NadA containing strain of *N. lactamica* or a genetically modified, but wild type-equivalent control strain. These challenge volunteers will be admitted for 4.5 days observation following inoculation and will then be discharged with strict infection control rules. Bedroom contacts of the challenge volunteers will also be enrolled as contact volunteers. Safety, colonisation, shedding, transmission and immunogenicity will be assessed over 90 days after which carriage will be terminated with antibiotic eradication therapy.

Ethics and dissemination

This study has been approved by the Department for Environment, Food and Rural Affairs (DEFRA) [1] and South Central Oxford A Research Ethics Committee reference: 18/SC/0133. Findings will be published in peer-reviewed open access journals as soon as possible.

For peer review only

STRENGTHS AND LIMITATIONS OF THIS STUDY

- This human challenge study using a genetically modified organism will provide insight into the role of a specific bacterial antigen in nasopharyngeal carriage and immunity, and provide a novel means to test the herd-immunity potential of vaccines
- Safety is the first priority and has been considered at all points of the study design with extensive pre-clinical testing, a period of admission for close observation following inoculation and stringent infection control rules throughout the study
- The use of environmental sampling and regular contact volunteer sampling will provide new information regarding the shedding and transmission of respiratory tract organisms
- The planned inoculum dose is based on previous studies with wild type *N. lactamica* and may not be the optimal dose to achieve colonisation with the genetically modified strains
- The low number of participants may be insufficient to prove an effect of the expression of NadA on colonisation so further research may be required

For peer review only

INTRODUCTION

Neisseria lactamica and *Neisseria meningitidis*

Neisseria lactamica and *Neisseria meningitidis* are Gram negative diplococci which both colonise the human nasopharynx. *Neisseria lactamica* is non-pathogenic, non-encapsulated and lactose fermenting and is a common commensal, particularly in young children [2 3]. In contrast *N. meningitidis* expresses polysaccharide capsule and although it usually colonises asymptomatically, it can in a minority of colonised individuals, cause invasive disease [4 5]. Due to recombination events, the organism exists in multiple clonal forms, with specific clonal complexes being characteristically associated with invasive disease[6]. Invasive meningococcal disease remains a significant global cause of morbidity and mortality with sporadic disease and small outbreaks throughout the world and significant epidemics occurring in the meningococcal belt of sub-Saharan Africa. (Harrison 2009)

Carriage of *N. lactamica* and *N. meningitidis*

Of note, *N. lactamica* appears to provide commensal-related protection against meningococcal disease. Age-specific rates of *N. meningitidis* carriage and disease are inversely proportional to carriage of *N. lactamica* [7-9]. The highest rate of natural carriage of *N. lactamica* occurs in infants. This then wanes in toddlers and older children and by adolescence carriage is approximately 1% [2 7]. Carriage of *N. meningitidis* is low in infants, increasing gradually throughout childhood and peaking in adolescence with the highest rates of carriage seen in teenagers and University students [10].

The mechanism of this epidemiological relationship is as yet undetermined. It is probably not due to cross-protective antibody production; the early years of life associated with high rates of *N. lactamica* carriage predate the development of natural bactericidal meningococcal antibodies [4]. Other postulated mechanisms

For peer review only

include microbial competition, innate immune responses triggered by *N. lactamica* colonisation and cross-reactive non-humoral acquired immunity [11 12].

Human challenge with *Neisseria lactamica*

A controlled human infection model of *N. lactamica* colonisation has been utilised to investigate the mechanism of this natural effect. Previous studies have shown that human challenge with wild type *N. lactamica* is safe and can induce long standing colonisation. Over 350 healthy adult volunteers have been experimentally nasally inoculated with wild type *N. lactamica* in previous studies. The colonisation fraction (the percentage of individuals who are colonised after challenge) was 35-65% [11 12]. Colonisation resulted in the development of humoral immunity to *N. lactamica* but no evidence of cross reactive bactericidal antibodies to *N. meningitidis*. Some cross-reactive opsonophagocytic antibody production occurred but was rather weak. [12]. In another large study, successful colonisation with *N. lactamica* was associated with the displacement of pre-existing meningococcal carriage, and inhibition of acquisition of *N. meningitidis* [11] supporting the role of *N. lactamica* carriage in protection from meningococcal carriage and therefore disease.

Meningococcal vaccines

Glycoconjugate vaccines directed against capsular antigens for serogroups C, A, W-135 and Y have been in use globally for several years. These have had dramatic effects on disease incidence, which is probably mostly due to herd protection conferred by vaccine-induced modification of colonisation reducing inter-host transmission [13 14]. Recent vaccine developments include a new subcapsular vaccine, 4CMenB (Bexsero), which induces bactericidal antibodies against a range of strains, including serogroup B, and protects vaccinated infants against disease [15]. In view of the importance of carriage-reduction for herd immunity, a large prospective randomised study was done to measure this, but the effect of Bexsero on carriage of *N. meningitidis* was found to be relatively modest and delayed until 3 months after vaccination [16], with no evidence of an effect on carriage of the serogroup B organisms carried by the participants.

For peer review only

More rapidly effective and longer lasting vaccines are required, particularly to halt transmission during epidemics in the meningitis belt of sub-Saharan Africa. Successful future vaccines should maximise herd immunity by targeting carriage and transmission. The development of such vaccines requires a greater understanding of mucosal immune mechanisms and the specific antigens involved in colonisation.

The meningococcal antigen NadA

In this human challenge study volunteers will receive intranasal inoculation with a genetically modified (GM) strain of *N. lactamica* expressing the meningococcal antigen NadA. This antigen is being used because it is well defined, and one of the 4 strongly immunogenic components of the Bexsero vaccine. Bexsero and has been demonstrated to be immunogenic in terms of generating serum bactericidal antibodies against *N. meningitidis* strains that express NadA [17] and moderately effective in reducing acquisition of nasopharyngeal carriage of *N. meningitidis* over the course of 12 months after vaccination [16]. NadA expression by *N. lactamica* may induce systemic and mucosal immunity to NadA. When studied alongside a control strain, use of a GMO *N. lactamica* expressing NadA could permit advanced study of the mechanisms underlying mucosal immunity and carriage-reduction. Furthermore, a GMO *N. lactamica* expressing NadA might exhibit enhanced protection against carriage of virulent *N. meningitidis*.

Rationale for this study

The rationale for this study is to pilot the use of the transformed commensal *N. lactamica* as an experimental medicine tool to study immunity to meningococcal antigens in humans, and to investigate the potential utility of genetically transformed commensals as tools to investigate the efficacy of vaccines to prevent colonisation of organisms expressing specific antigens. Finally, expression of NadA might lead to increased efficiency of harmless colonisation by *N. lactamica* and prompt the development of this GMO as a bacterial medicine.

METHODS AND ANALYSIS

Study overview

This is a prospective controlled human challenge study in which challenge volunteers will be inoculated intranasally with *Neisseria lactamica* genetically modified to express NadA (the intervention strain) or a control genetically modified strain. An inoculum dose of 10^5 CFU will be used for both strains. Following inoculation, challenge volunteers will be admitted to Southampton National Institute for Health Research Clinical Research Facility (NIHR CRF) for 4.5 days. A further group of volunteers, who are close contacts of the participants will be enrolled to detect transmission of the inoculated strains. Safety parameters, colonisation, shedding, transmission and immunogenicity will be assessed during the admission period and over a follow up period of approximately 3 months. Colonisation will be terminated with antibiotic eradication therapy on Day 90, for all challenge and contact volunteers.

Study objectives

The objectives of this study are to establish the safety and NadA-specific immunogenicity of nasal inoculation with the intervention strain of GM *N. lactamica* and to assess subsequent shedding and transmission. A further objective is to assess the efficacy of ciprofloxacin eradication therapy. These objectives and the study endpoints are summarised in Table 1 below:

For peer review only

	Objectives	Endpoints
Co-primary objectives	To establish the safety of nasal inoculation of healthy volunteers with a genetically modified strain of *Neisseria lactamica* expressing NadA	Occurrence of unsolicited adverse events within the study period
		Occurrence of serious adverse events within the study period
	To assess the NadA specific immunity in healthy volunteers following nasal inoculation with *Neisseria lactamica* expressing NadA	Rise in serological specific IgG titre (anti-NadA) comparing day 0 versus days 14 to 90 comparing volunteers colonised by one of the two GMOs
		Rise in mucosal specific antibody titre comparing day -5 versus days 3 to 90 and comparing volunteers colonised with the two GMOs
		Change in nasal cytokine profile comparing day 0 versus days 3 to 90 and comparing volunteers colonised with the two GMOs
Secondary objectives	To assess the shedding of genetically modified *Neisseria lactamica* following nasal inoculation	Culture of GM *N. lactamica* from environmental samples – comparing intervention and control groups
	To assess the transmission of genetically modified *Neisseria lactamica* to bedroom contacts of inoculated volunteers	Culture of GM *N. lactamica* from throat swabs taken from contact volunteers from day 4 until day 90 – comparing intervention and control groups
	To assess the efficacy of a single dose of Ciprofloxacin in eradicating carriage of genetically modified *Neisseria lactamica*	Culture of GM *N. lactamica* from throat swabs taken at the eradication visit in comparison to post-eradication visit in challenge and contact volunteers

Table 1 – Objectives and Endpoints

For peer review only

Genetically modified *Neisseria lactamica*

The intervention strain

The intervention strain has been transformed by the integration of the *N. meningitidis* gene *nadA* (NEIS1969), leading to expression of NadA. The NadA protein is a member of the type V autotransporter family of outer membrane proteins, and in *N. meningitidis* is associated with an increased level of adhesion to and invasion of human epithelial cell lines. The presence of the *nadA* gene in the genome is associated with hypervirulent lineages of *N. meningitidis*, but NadA surface expression has not been shown to be causal for increased virulence.

The control strain

The control strain has been genetically modified in exactly the same way as the intervention strain, except that it does not contain the coding sequence for the *nadA* gene. In terms of gene content and behaviour in the laboratory, this strain is extremely similar to wild type. Using this strain as a control inoculum is superior to using the wild type strain as the changes made to the genetic architecture and gene regulation are identical to the intervention strain apart from the insertion of *nadA*.

Pre-clinical safety data

Both strains have been demonstrated to remain acutely susceptible to killing by normal human serum and retain sensitivity to the antibiotics used clinically to treat meningococcal disease (rifampicin, ciprofloxacin and ceftriaxone). Pre-clinical testing [1] has shown that the NadA autotransporter is functionally expressed in the intervention strain, the NadA protein is strongly immunogenic in the context of expression in *N. lactamica* and that expression of NadA does not significantly increase pathogenicity of the commensal in a murine model of infection. Neither strain has an increased propensity to become transformed by exogenous sources of DNA, which might otherwise allow it to acquire virulence factors such as an extracellular capsule, as compared to the wild type strain.

For peer review only

Quality assessment and control

Preparation, storage and monitoring of the challenge strains will be carried out to GMP-like standards at the University of Southampton. The dose and purity of the inoculum will be determined after inoculation for quality assessment.

The inoculum dose

Based on the previous *N. lactamica* human challenge studies it is estimated that 50% of volunteers will be colonised 1-2 weeks after inoculation at this dose (Evans, 2011). Fifty per cent (50%) has been chosen as an acceptable colonisation rate because it is below a “saturating” dose and therefore avoids the difficulties of interpretation of a challenge dose that is much higher than physiologically appropriate.

Study volunteers

Challenge volunteers

Healthy volunteers aged 18-45 years will be recruited and challenged until 11 volunteers in each group are colonised with GM *N. lactamica* at day 14 or up to a maximum of 22 inoculated volunteers in each group.

Contact volunteers

Contact volunteers are bedroom contacts of challenge volunteers, defined as individuals who share a bedroom on at least one occasion during the study period. A maximum of one contact volunteer may be recruited per challenge volunteer and contact volunteers must give informed consent prior to inoculation of the corresponding challenge volunteer. Bedroom contacts who are under 18 or who are immunocompromised will be excluded from participation, as will their corresponding challenge volunteer.

Eligibility criteria

We will not recruit from vulnerable groups such as those with impaired capacity. Those with close contact with potentially vulnerable people such as small children

For peer review only

and immunocompromised individuals will be excluded. Specific inclusion and exclusion criteria can be found in Supplementary table 1.

Infection control agreement

Both challenge and contact volunteers must provide written infection control agreement prior to enrolment, which will include agreement to have no other bedroom contacts during the study period. Details of the infection control requirements can be found in Supplementary table 2.

Study setting

The challenge procedure, admission and follow up visits will take place in the NIHR CRF at University Hospital Southampton NHS Foundation Trust.

Recruitment

Participants will be recruited via a variety of media including ethically approved adverts displayed within the hospital, on Southampton NIHR CRF websites, social media and circulated literature, the Southampton CRF database of healthy volunteers, presentations and press releases. Individuals who express an interest will be sent a volunteer information sheet. Volunteers will be offered reimbursement for their time, travel and inconvenience.

Study timeline

Challenge and contact volunteers will be enrolled from the date of screening, up to 90 days prior to the challenge procedure, until day 92 post challenge. The duration of volunteer participation will therefore be up to approximately 6 months. An overview of the study timeline is shown in Figure 1 below. Details of study procedures are shown in Supplementary table 3 (Challenge volunteers) and Supplementary table 4 (Contact volunteers).

Figure 1: Study timeline

For peer review only

Screening

Potential challenge and contact volunteers will be invited to separate screening visits up to 90 days prior to challenge. At these screening visits they will be fully informed of all aspects of their involvement in the study, be given an opportunity to ask questions, to give informed consent and to undergo a medical screening to determine eligibility. Challenge volunteers will be asked to complete a pre-consent questionnaire to ensure their understanding of the study and their medical history will be confirmed with their GP. The infection control guidelines (see Supplementary table 2) will be explained to all volunteers and they will be asked to complete a questionnaire to confirm their understanding of these guidelines, and to sign an agreement to follow these guidelines throughout the study period. Challenge volunteers will attend a pre-challenge visit the week prior to their challenge to ensure that they remain eligible.

First volunteers

For each GM strain the first volunteers will be challenged individually and then in pairs with a safety review after volunteers 1, 3 and 5. Further volunteers will be challenged in groups of a maximum of 5.

Challenge

Challenge volunteers will be admitted to a designated area of the NIHR CRF on the morning of their challenge procedure. Ongoing informed consent and eligibility will be confirmed and clinical samples will be taken for baseline immunology.

The inoculum will be prepared from frozen stocks and will be administered by a study doctor following study-specific standard operating procedures. The challenge will take place in an environmental chamber within the CRF. The challenge volunteer will be positioned supine with neck extended and breathing normally through their mouth. 0.5ml of inoculum will be administered slowly from a pipette into each nostril. The residual inoculum will be analysed to confirm the administered dose and purity. Public Health Southampton will be informed of all participants who have been challenged with the GMOs.

For peer review only

Admission

During admission, challenge volunteers will have access to an individual bedroom, shared bathroom facilities and a shared recreational area. Clinical observations and any symptoms will be recorded approximately every 4 hours and a study doctor will review volunteers twice a day. Clinical and environmental samples will be taken as detailed in table 2 below to assess safety, colonisation, immunogenicity and shedding.

	Day 0	Day 1	Day 2	Day 3	Day 4
Vital signs	Pre inoculation then 4 hourly	4 hourly	4 hourly	4 hourly	4 hourly
Review of adverse events	4 hourly	4 hourly	4 hourly	4 hourly	4 hourly
Medical review	x 2	x 2	x 2	x 2	x 2
Pregnancy test (females only)	+				
Review eligibility	+				
Inoculation	+				
Throat swab (culture)	+		+	+	+
Throat swab (microbiome)	+			+	
Nasal wash				+	
Nasosorption test	+			+	
Saliva sample	+			+	
Environmental samples		+	+	+	+
Safety bloods (ml)	8			8	
Immunological blood tests (ml)	70				

Table 2 – Study procedures during admission

Prior to discharge of the challenge volunteer, the contact volunteer will attend to confirm ongoing consent and eligibility and the infection control procedures will be reiterated to both challenge and contact volunteers.

Follow up

Following challenge volunteer discharge, volunteers will be monitored for adverse events, colonisation, shedding, transmission and immunogenicity as detailed in in

For peer review only

Supplementary table 3 (Challenge volunteers) and Supplementary table 4 (Contact volunteers).

Adverse events

Adverse events will be monitored at each follow up visit. In addition to this volunteers will be encouraged to contact the study team at any point during the study in the event any symptoms develop.

Colonisation

Colonisation will be assessed by culture of throat swabs and nasal washes. Colonisation density will be estimated by qPCR and comparison will be made between the intervention and control groups.

Shedding

Shedding of GM *N. lactamica* from inoculated challenge volunteers will be assessed by microbiological analysis of environmental samples. Comparison of shedding will be made between the intervention and control challenge volunteers. Environmental sampling will include culture and PCR of face mask samples and air samples taken within an environmental chamber during aerosol producing activities.

A challenge volunteer in the intervention group will be considered to have increased shedding at a particular time point if they have a 10-fold increase in shedding in comparison to the average shedding seen at the same time point in colonised control group volunteers to date. This is a nominal figure agreed with the statutory authority (UK Department for the Environment, Food and Rural Affairs) because of the unpredictable scale and frequency of this event which will not permit a prospective, statistically-based assessment of potentially hazardous release to the environment. If increased shedding is seen at any point from the Day 14 visit then the volunteer will be asked to attend as soon as possible for an additional shedding check visit. If increased shedding is seen at two consecutive visits this will be considered enhanced shedding.

For peer review only

Transmission

Transmission will be assessed by culture and PCR of throat swabs from contact volunteers. Comparison will be made between the intervention and control groups.

Immunogenicity

Mucosal and systemic immunogenicity will be investigated. Saliva and nasal secretions will be collected for assessment of mucosal immunogenicity and blood samples for systemic humoral and cellular responses.

Eradication

Antibiotic eradication therapy will be given to all challenge and contact volunteers with a throat swab to confirm successful eradication after a maximum of 48 hours. Standard eradication will be given to all volunteers at Day 90 (regardless of colonisation status) with a confirmatory throat swab on Day 92. Eradication therapy may be given at an earlier time point under specific circumstances.

Triggered eradication may be given to volunteers at any time point due to:

- Safety concerns in the challenge volunteer or corresponding contact volunteer, at the discretion of the study team
- Enhanced shedding from the challenge volunteer
- Study withdrawal for any other reason

If eradication is triggered for a challenge or contact volunteer then their corresponding challenge or contact volunteer (if applicable) will receive eradication therapy on the same day and both volunteers will be withdrawn from the study.

In addition to this, contact volunteers found to be colonised with GM *N. lactamica* at any point may receive early eradication therapy, as ongoing colonisation of contact volunteers is not required to fulfil the study objectives. In this case the corresponding challenge volunteer will not receive eradication therapy and both will continue in the study as planned.

A single dose of 500 mg ciprofloxacin will be taken under supervision of the study team. All female volunteers will have a pregnancy test prior to eradication. In the

For peer review only

event of a positive pregnancy test, alternative eradication therapy will be used – Rifampicin 600 mg bd for 48 hours.

Both Rifampicin and Ciprofloxacin, as oral antibiotics, have been shown to be effective in eradicating carriage of *N. meningitidis* [18], and are regularly used as post exposure prophylaxis [19]. Both GM strains are also sensitive to these antibiotics.

Study holding rules

An independent external safety committee will review the safety aspects of the study on a regular basis and in the event of any significant safety concerns. Colonisation, shedding, transmission and clinical parameters will be closely monitored throughout the study. In the event of a study holding criterion being met the study will be paused for a safety review. No further volunteers will be challenged until the data have been reviewed by the external safety committee and study continuation approved.

Enhanced colonisation

The expression of NadA by the intervention strain of GM *N. lactamica* is expected to be associated with either an increase or a decrease in colonisation frequency or density compared to wild type. Colonisation rate and density estimation will be monitored but an increase in colonisation alone will not trigger a study pause unless associated with sustained enhanced shedding, transmission or safety concerns.

Enhanced shedding

Enhanced shedding triggering early eradication in 3 or more of the first 5 volunteers to receive the intervention strain or in >50% of ongoing challenge volunteers in the intervention group will trigger a study pause.

Enhanced transmission

Transmission of either strain of GM *N. lactamica* to 3 of the first 5 or >50% of ongoing contact volunteers will trigger a study pause.

GM N. lactamica disease

If antibiotic treatment (IV ceftriaxone or IV chloramphenicol) is given to any volunteer due to possible GM *N. lactamica* disease then a study pause will be triggered.

For peer review only

Sample size

We are aiming to achieve colonisation in 10 challenge volunteers for each of the GM strains. This is based on a previous experimental *N. lactamica* challenge study, which showed a significant rise in serological antibody titre against *N. lactamica* over 2 weeks [12]. This gave SDs on a log-10 scale of 0.11 for IgA saliva and 0.26 for serum total IgG. For this study, using the SD of 0.26 we will be able to confirm a 4 fold rise of anti-NadA with 10 carriers of *N. lactamica* expressing NadA with 90% power using analysis of variance.

Allowing for a drop out rate of approximately 10%, we will therefore recruit challenge volunteers until we have 11 individuals colonised for each group up to a maximum of 22 volunteers for each group. Estimating a colonisation fraction of 50%, approximately 44 individuals will be enrolled as challenge volunteers. A maximum of one contact volunteer will be enrolled per challenge volunteer.

Patient and Public Involvement

A PPI group was consulted during the early stages of study design to discuss the implications of human challenge with a genetically modified organism. An important suggestion arising from this consultation was to seek information about the potential for spread of infection which we have discussed further with PHE experts and DEFRA. As a result of these discussions, our protocol includes close monitoring of environmental shedding and transmission to sleeping partners with specific action points in the event that there is evidence of enhanced shedding into the environment. Suggestions from the PPI consultation were also used in the design of the volunteer information sheet.

In addition, formal and informal feedback from volunteers involved in other human challenge trials in the NIHR Clinical Research Facility Southampton has been used to refine the design of this study and preparation of the admission area.

Participants in this study will be provided with a lay summary of the results once available.

For peer review only

ETHICS AND DISSEMINATION

As this study involves the deliberate release of genetically modified bacteria into the community it has been considered and approved by the responsible government ministry - the Department for Environment, Food and Rural Affairs [1].

It has also been reviewed and approved by South Central Oxford A Research Ethics Committee (SC/18/0113) and by the UK Health Research Authority (IRAS ID 235090). Results will be published in peer-reviewed journals once available.

For peer review only

DISCUSSION

Human challenge with a genetically modified organism – safety considerations

This study will result in the deliberate release of two genetically modified organisms (GMOs). One previous study has been published in which volunteers were deliberately inoculated with a GMO that has therefore potentially been released into the general population. In that study, carried out in Sweden, a genetically modified attenuated *Bordetella pertussis* strain was constructed as a vaccine candidate. This was administered nasally, in order to mimic natural infection without inducing disease and volunteers were subsequently followed up as outpatients[20].

In the United Kingdom the deliberate release of a GMO requires DEFRA approval. This protocol has therefore been reviewed by DEFRA who have considered the potential for colonisation of other members of the general population, and have given approval of the study.

During the design of this study, our priority has been to ensure the safety of the volunteers to limit the potential for transmission to close contacts of the volunteers, study team members and the wider population. A number of safety considerations have been incorporated into the protocol and an independent external safety committee will review the safety aspects of the study on a regular basis.

Safety of GM *N. lactamica*

N. lactamica is a non-virulent commensal organism and there have been no safety concerns in previous challenge studies with the wild type organism. There is no evidence to suggest that the genetically modified strains will be more likely than wild type to cause invasive disease, as the organisms are non-capsulate and highly susceptible to killing by human serum. Pre-clinical work has indicated that the GMOs are stable, do not undergo recombination events at higher frequency than wild type

For peer review only

and are non-virulent when inoculated into mice. We therefore consider that the likelihood of the GMO causing any disease is extremely low.

Safety of challenge and contact volunteers

For each strain, the first five challenges will be staggered with a safety review between challenges. All challenged volunteers will be admitted to Southampton NIHR CRF for close observation for 4.5 days following challenge. The period of risk of development of invasive meningococcal disease is the first 48 hours following acquisition so in the unlikely event of any volunteer developing symptoms it would be expected to occur within this period of admission. The NIHR CRF is funded and staffed to allow the delivery of higher risk experimental studies and is located within an NHS hospital so study nurses will be immediately available, study doctors will be contactable and able to attend and full NHS clinical services will be present within the same building if required. Following discharge all volunteers will be monitored regularly for adverse events and will be given a 24 hour phone number to contact the study team.

Minimising onward transmission

Transmission occurs through close contact and previous studies looking at the transmission of *N. lactamica* and *N. meningitidis* suggest that household members, and in particular bedroom-sharers of colonised individuals are those at highest risk of acquisition of carriage [21-23]. Bedroom sharers of challenge volunteers are therefore the most relevant community members to screen for transmission and so will give informed consent and will be enrolled as contact volunteers for this purpose. Potential challenge or contact volunteers with household members or other close contacts who may be at increased risk of acquisition of carriage or of *N. lactamica* disease will be excluded from the study.

Other infection control measures include the use of PPE, strict infection control guidelines, and close monitoring of shedding and transmission. These measures have been designed to limit the potential onward transmission of the inoculated bacteria to study team members, vulnerable individuals and to the general

For peer review only - http://bmjopen.bmj.com/site/about/guidelines.xhtml

population. In addition all volunteers will receive eradication therapy prior to study completion, regardless of their colonisation status.

The benefit of a human challenge model

A greater understanding of the mucosal immune mechanisms of protection from colonisation is essential for the development and evaluation of new vaccines, specifically ones targeting colonisation and transmission. The most direct and effective way to achieve this is experimental controlled human infection. This model can be used to investigate in detail components of mucosal and systemic immunity activated in real time following infection with a defined antigen. Also, this model could be used to investigate vaccine efficacy. For example, healthy volunteers who have received a study vaccine could then be challenged with a defined organism expressing constituent antigens. Monitoring carriage of the challenge bacterium over time would then provide information of the efficacy of the vaccine in the prevention of colonisation. Experimental human challenge with pathogens of interest such as *N. meningitidis* would be potentially hazardous and therefore raise significant ethical and logistical issues. The use of a harmless commensal organism that has been transformed to express specific antigens could be a safe and effective alternative.

N. lactamica is an appropriate organism to be transformed for this purpose. It is a well-studied and characterised commensal organism, which is known to exclusively colonise the human nasopharynx. It is genetically very similar to *N. meningitidis*, sharing approximately 67% of the genes believed to be associated with meningococcal virulence [24]. Despite this, *N. lactamica* is known to be non-virulent and has been used safely in previous human challenge studies.

N. lactamica is the only member of the genus *Neisseria* which is able to ferment lactose due to the activity of β-D-galactosidase coded for by the gene *lacZ.* This causes colonies to grow blue on the chromogenic substrate 5-bromo-4-chloro-3-indolyl β-D-galactopyranoside (X-gal). This characteristic has been utilised in our study; both of our GM strains have been derived from a *lacZ* deficient strain of *N. lactamica* Y92-1009 (Δ*lacZ*), which grows as white colonies on X-gal-containing medium. During the transformation process *lacZ* has been re-integrated as a marker

of successful transformation, thus allowing screening for successful transformants on the basis of blue/white colony formation on X-gal-containing medium. This has been done to completely avoid the use of genes coding for resistance to antibiotics and to eliminate the risk of our challenge experiment disseminating antimicrobial resistance genes into the nasopharyngeal microbiome.

The meningococcal antigen NadA has been chosen as the specific antigen for this study. NadA is a component of the Bexsero vaccine and is known to be potently immunogenic so successful colonisation is likely to induce the production of specific anti-NadA antibodies. Indeed, in a murine nasal challenge model, wherein genetically modified *Streptococcus gordonii* expressing meningococcal NadA was used to inoculate mice, colonised subjects produced systemic anti-NadA bactericidal antibodies and localised anti-NadA IgA [25]. The *nadA* gene is associated with hypervirulent strains of *N. meningitidis* and was present in 50% of strains isolated from cases of meningococcal disease [26]. NadA has a role in increased adhesion and invasion into human epithelial cells [27] so NadA expression may therefore increase the ability of *N. lactamica* to colonise the nasopharynx. However *nadA* is absent from some virulent strains and the majority of non-virulent strains of *N. meningitidis*, which may limit the potential for cross-reactive immunity [26 28]. In addition, as NadA is so potently immunogenic, expression may in fact reduce the duration of colonisation due to enhanced clearance.

Once this human challenge model has been shown to be safe and effective it could potentially be used to study other meningococcal antigens, or indeed antigens from other respiratory mucosal pathogens.

The potential for use as a bacterial medicine

Carriage of wild type *N. lactamica* appears to be protective against meningococcal disease, at least partly due to physical competition. The modification of *N. lactamica* to express an adhesin such as NadA could plausibly improve the colonisation fraction or colonisation duration.

For peer review only

Colonisation with *N. lactamica* has been shown to result in some cross-reactive acquired immunity to *N. meningitidis*, but this is insufficient to be fully protective [12]. Genetic modification of *N. lactamica* to express a meningococcal antigen known to be potently immunogenic may lead to the production of anti-meningococcal serum bactericidal antibodies (SBA).

If successful, these improvements in the protective effect of induced colonisation with *N. lactamica* may lead to its potential use as a bacterial medicine.

Conclusion

The successful and safe colonisation of healthy volunteers with genetically modified strains of *N. lactamica* will pave the way for further challenge studies involving transformants which express other meningococcal antigens, and potentially antigens expressed by other pathogens. These challenge models will lead to a greater understanding of mucosal immune responses to colonisation and infection, provide a platform for the development and assessment of improved vaccines, and may lead to the development of novel bacterial medicines.

AUTHORS' CONTRIBUTIONS

The study was designed by DG, JRL and RCR with input from all authors. The first draft of this manuscript was prepared by DG and all authors then contributed to editing and approved the final version of this manuscript.

ACKNOWLEDGEMENTS

We would like to acknowledge the input of a Public and Patient Involvement group in the early design stage of this study.

For peer review only

FUNDING STATEMENT

This work will be supported by the Medical Research Council (Grant MR/N026993/1) "Pathfinder: Experimental Human Challenge with Genetically Modified Commensals to Investigate Respiratory Tract Mucosal Immunity and Colonisation" and the MRC Confidence in Concept Award, with additional funding from Experimental Medicine by the National Institute for Health Research through support from the Southampton NIHR CRF and the Biomedical Research Centre. The development of the technology underpinning the genetic modification was funded by the Medical Research Council (A genetically modified nasopharyngeal commensal as a platform for bacterial therapy, MR/N013204/1).

COMPETING INTERESTS STATEMENT

JRL and RR declare a potential conflict of interest: The patent WO2017103593-A1 `New modified *Neisseria lactamica* transformed with recombinant DNA encoding heterologous protein, used for e.g. prophylactic treatment of pathogenic infection, preferably meningococcal infection`, is assigned to the University of Southampton, with Dr JR Laver, and Professor RC Read as inventors.

For peer review only

REFERENCES

1. Laver JR, Read RC. Genetically Modified Organisms: University of Southampton (17/R50/01) 2017 [Available from: https://www.gov.uk/government/publications/genetically-modified-organisms-university-of-southampton-17r50012017.
2. Bennett JS, Griffiths DT, McCarthy ND, et al. Genetic diversity and carriage dynamics of Neisseria lactamica in infants. *Infection and immunity* 2005;73(4):2424-32. doi: 10.1128/IAI.73.4.2424-2432.2005
3. Liu G, Tang CM, Exley RM. Non-pathogenic Neisseria: members of an abundant, multi-habitat, diverse genus. *Microbiology (Reading, England)* 2015;161(7):1297-312. doi: 10.1099/mic.0.000086
4. Trotter CL, Gay NJ, Edmunds WJ. The natural history of meningococcal carriage and disease. *Epidemiol Infect* 2006;134(3):556-66. doi: 10.1017/S0950268805005339
5. Stephens DS, Greenwood B, Brandtzaeg P. Epidemic meningitis, meningococcaemia, and Neisseria meningitidis. *Lancet (London, England)* 2007;369(9580):2196-210. doi: 10.1016/S0140-6736(07)61016-2
6. Read RC. Neisseria meningitidis; clones, carriage, and disease. *Clinical microbiology and infection : the official publication of the European Society of Clinical Microbiology and Infectious Diseases* 2014;20(5):391-95. doi: 10.1111/1469-0691.12647
7. Gold R, Goldschneider I, Lepow ML, et al. Carriage of Neisseria meningitidis and Neisseria lactamica in infants and children. *The Journal of infectious diseases* 1978;137(2):112-21.
8. Cartwright KA, Stuart JM, Jones DM, et al. The Stonehouse survey: nasopharyngeal carriage of meningococci and Neisseria lactamica. *Epidemiol Infect* 1987;99(3):591-601.
9. Olsen SF, Djurhuus B, Rasmussen K, et al. Pharyngeal carriage of Neisseria meningitidis and Neisseria lactamica in households with infants within areas with high and low incidences of meningococcal disease. *Epidemiology and infection* 1991;106(3):445-57.
10. Christensen H, May M, Bowen L, et al. Meningococcal carriage by age: a systematic review and meta-analysis. *The Lancet Infectious diseases* 2010;10(12):853-61. doi: 10.1016/S1473-3099(10)70251-6
11. Deasy AM, Guccione E, Dale AP, et al. Nasal Inoculation of the Commensal Neisseria lactamica Inhibits Carriage of Neisseria meningitidis by Young Adults: A Controlled Human Infection Study. *Clinical infectious diseases : an official publication of the Infectious Diseases Society of America* 2015;60(10):1512-20. doi: 10.1093/cid/civ098 [published Online First: 2015/03/31]
12. Evans CM, Pratt CB, Matheson M, et al. Nasopharyngeal colonization by Neisseria lactamica and induction of protective immunity against Neisseria meningitidis. *Clinical infectious diseases : an official publication of the Infectious Diseases Society of America* 2011;52(1):70-7. doi: 10.1093/cid/ciq065 [published Online First: 2010/12/15]

For peer review only

13. Maiden MC, Ibarz-Pavón AB, Urwin R, et al. Impact of meningococcal serogroup C conjugate vaccines on carriage and herd immunity. *The Journal of infectious diseases* 2008;197(5):737-43. doi: 10.1086/527401
14. Trotter CL, Maiden MC. Meningococcal vaccines and herd immunity: lessons learned from serogroup C conjugate vaccination programs. *Expert review of vaccines* 2009;8(7):851-61. doi: 10.1586/erv.09.48
15. Parikh SR, Andrews NJ, Beebeejaun K, et al. Effectiveness and impact of a reduced infant schedule of 4CMenB vaccine against group B meningococcal disease in England: a national observational cohort study. *Lancet (London, England)* 2016;388(10061):2775-82. doi: 10.1016/S0140-6736(16)31921-3
16. Read RC, Baxter D, Chadwick DR, et al. Effect of a quadrivalent meningococcal ACWY glycoconjugate or a serogroup B meningococcal vaccine on meningococcal carriage: an observer-blind, phase 3 randomised clinical trial. *Lancet (London, England)* 2014;384(9960):2123-31. doi: 10.1016/S0140-6736(14)60842-4
17. Read RC, Dull P, Bai X, et al. A phase III observer-blind randomized, controlled study to evaluate the immune response and the correlation with nasopharyngeal carriage after immunization of university students with a quadrivalent meningococcal ACWY glycoconjugate or serogroup B meningococcal vaccine. *Vaccine* 2017;35(3):427-34. doi: 10.1016/j.vaccine.2016.11.071
18. Gaunt PN, Lambert BE. Single dose ciprofloxacin for the eradication of pharyngeal carriage of Neisseria meningitidis. *J Antimicrob Chemother* 1988;21(4):489-96.
19. Fraser A, Gafter-Gvili A, Paul M, et al. Antibiotics for preventing meningococcal infections. *The Cochrane database of systematic reviews* 2006(4):CD004785. doi: 10.1002/14651858.CD004785.pub3 [published Online First: 2006/10/21]
20. Thorstensson R, Trollfors B, Al-Tawil N, et al. A phase I clinical study of a live attenuated Bordetella pertussis vaccine--BPZE1; a single centre, double-blind, placebo-controlled, dose-escalating study of BPZE1 given intranasally to healthy adult male volunteers. *PloS one* 2014;9(1) doi: 10.1371/journal.pone.0083449
21. Simmons G, Martin D, Stewart J, et al. Carriage of N. lactamica in a population at high risk of meningococcal disease. *Epidemiology and infection* 2000;125(1):99-104.
22. Cartwright KA, Stuart JM, Robinson PM. Meningococcal carriage in close contacts of cases. *Epidemiology and infection* 1991;106(1):133-41.
23. Kristiansen BE, Tveten Y, Jenkins A. Which contacts of patients with meningococcal disease carry the pathogenic strain of Neisseria meningitidis? A population based study. *BMJ (Clinical research ed)* 1998;317(7159):621-25.
24. Snyder LA, Saunders NJ. The majority of genes in the pathogenic Neisseria species are present in non-pathogenic Neisseria lactamica, including those designated as 'virulence genes'. *BMC genomics* 2006;7:128. doi: 10.1186/1471-2164-7-128
25. Ciabattini A, Giomarelli B, Parigi R, et al. Intranasal immunization of mice with recombinant Streptococcus gordonii expressing NadA of Neisseria meningitidis induces systemic bactericidal antibodies and local IgA. *Vaccine* 2008;26(33):4244-50. doi: 10.1016/j.vaccine.2008.05.049

For peer review only

26. Comanducci M, Bambini S, Caugant DA, et al. NadA diversity and carriage in Neisseria meningitidis. *Infection and immunity* 2004;72(7):4217-23. doi: 10.1128/IAI.72.7.4217-4223.2004
27. Capecchi B, Adu-Bobie J, Di Marcello F, et al. Neisseria meningitidis NadA is a new invasin which promotes bacterial adhesion to and penetration into human epithelial cells. *Molecular microbiology* 2005;55(3):687-98. doi: 10.1111/j.1365-2958.2004.04423.x
28. Vogel U, Taha M-KK, Vazquez JA, et al. Predicted strain coverage of a meningococcal multicomponent vaccine (4CMenB) in Europe: a qualitative and quantitative assessment. *The Lancet Infectious diseases* 2013;13(5):416-25. doi: 10.1016/S1473-3099(13)70006-9

For peer review only

FIGURES:

Figure 1 – Study timeline

TABLES:

Table 1 – Objectives and endpoints

Table 2 – Study procedures during admission

SUPPLEMENTARY TABLES:

Supplementary table 1 – Eligibility criteria

Supplementary table 2 – Infection control guidelines

Supplementary table 3 – Study timetable for challenge volunteers

Supplementary table 4 – Study timetable for contact volunteers

For peer review only

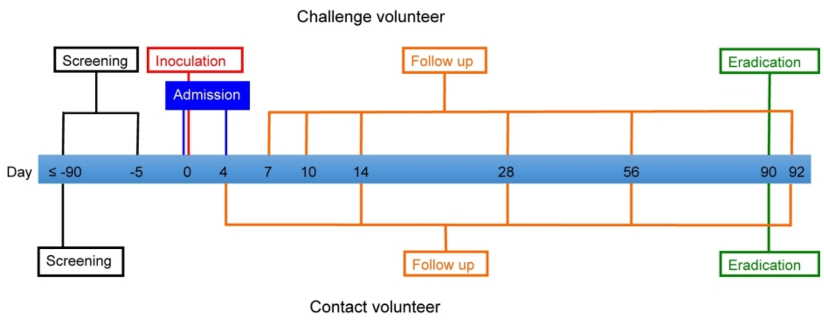

Study Timeline

190x107mm (300 x 300 DPI)

SUPPLEMENTARY TABLE 1: ELIGIBILITY CRITERIA

Inclusion criteria

Challenge volunteers	Contact volunteers
Healthy adults aged 18 to 45 years inclusive on the day of enrolment	Healthy adults aged 18 years or over on the day of enrolment
Fully conversant in the English language	
Able and willing (in the investigator's opinion) to comply with all study requirements	
Provide written informed consent to participate in the trial	
Provide written agreement to abide by infection control guidelines including agreement to abstain from intimate contact with any individual other than one declared and consented bedroom contact during the study period	
Provide written consent to allow the study team to discuss the volunteer's medical history with the General Practitioner	
Written informed contact volunteer consent provided by any bedroom contact	
Agreement to be admitted to Southampton NIHR-CRF for 4.5 days following inoculation	
For females only, willingness to practice continuous effective contraception (see below) during the study and a negative pregnancy test on the day(s) of screening and inoculation	For females only, willingness to practice continuous effective contraception (see below) during the study and a negative pregnancy test on the day of screening and challenge volunteer discharge
Agreement to take antibiotic eradication therapy according to the study protocol	
Able to correctly answer all questions in the pre-consent and infection control questionnaires	Able to correctly answer all questions in the infection control questionnaire
TOPS registration completed and no conflict found	

NIHR-CRF: National Health Institute for Health Research-Clinical Research Facility, TOPS: The Over-volunteering Prevention System

For peer review only

Effective contraception for female volunteers

Established use of oral, injected or implanted hormonal methods of contraception
Placement of an intrauterine device or intrauterine system
Total abdominal hysterectomy
Barrier methods of contraception (condom or occlusive cap with spermicide)
Male sterilisation if the vasectomised partner is the sole partner for the subject
True abstinence when this is in line with the preferred and usual lifestyle of the subject

For peer review only

Exclusion criteria

<table>
<tr><th>Challenge volunteers</th><th>Contact volunteers</th></tr>
<tr><td>Current active smokers defined as having smoked a cigarette or cigar in the last four weeks</td><td></td></tr>
<tr><td>N. lactamica or N. meningitidis detected on throat swab or nasal wash taken at screening or at the pre-challenge visit</td><td></td></tr>
<tr><td>Individuals who have a current infection at the time of inoculation</td><td></td></tr>
<tr><td colspan="2">Individuals who have been involved in other clinical trials involving receipt of an investigational product over the last 12 weeks or if there is planned use of an investigational product during the study period</td></tr>
<tr><td>Individuals who have previously been involved in clinical trials investigating meningococcal vaccines or experimental challenge with N. lactamica</td><td></td></tr>
<tr><td>Individuals who have received one or more doses of the meningococcus B vaccine Bexsero</td><td></td></tr>
<tr><td>Use of systemic antibiotics within the period 30 days prior to the challenge</td><td></td></tr>
<tr><td colspan="2">Any confirmed or suspected immunosuppressive or immune-deficient state, including HIV infection; malignancy, asplenia; recurrent, severe infections and chronic (more than 14 days) immunosuppressant medication within the past 6 months (topical steroids are allowed)</td></tr>
<tr><td>Use of immunoglobulins or blood products within 3 months prior to enrolment.</td><td></td></tr>
<tr><td>History of allergic disease or reactions likely to be exacerbated by any component of the inoculum</td><td></td></tr>
<tr><td colspan="2">Contraindications to the use of ciprofloxacin, specifically a history of epilepsy, prolonged QT interval, hypersensitivity to quinolones or a history of tendon disorders related to quinolone use</td></tr>
<tr><td colspan="2">Contraindications to the use of ceftriaxone, specifically hypersensitivity to any cephalosporins</td></tr>
<tr><td>Any clinically significant abnormal finding on clinical examination or screening investigations. In the event of abnormal test results, confirmatory repeat tests will be requested.</td><td></td></tr>
<tr><td colspan="2">Any other significant disease, disorder, or finding which may significantly increase the risk to the volunteer because of participation in the study, affect the ability of the volunteer to participate in the study or impair interpretation of the study data.</td></tr>
<tr><td colspan="2">Occupational, household or intimate contact with immunosuppressed persons, specifically HIV infection with a CD4 count <200 cells/mm3; asplenia; any malignancy, recurrent, severe infections and chronic (more than 14 days) immunosuppressant medication within the past 6 months (topical steroids are allowed)</td></tr>
<tr><td colspan="2">Occupational or household contact with children under 5 years or an older child with a tendency to co-sleep with the volunteer</td></tr>
<tr><td colspan="2">Pregnancy, lactation or intention to become pregnant during the study</td></tr>
<tr><td>Inability of the study team to contact the volunteer's GP to confirm medical history and safety to participate</td><td></td></tr>
</table>

For peer review only - http://bmjopen.bmj.com/site/about/guidelines.xhtml

SUPPLEMENTARY TABLE 2: INFECTION CONTROL GUIDELINES

During admission – challenge volunteers only:

- The volunteer must wear a surgical mask covering the nose and mouth at all times unless within their personal room, while showering or having respiratory samples taken or while outside in open air
- The volunteers are not allowed to enter the personal rooms of other volunteers
- The volunteer must wash his/her hands before leaving their personal room
- The volunteer is not allowed to leave the NIHR-CRF without permission of the clinical team
- Volunteers are allowed to leave the NIHR-CRF for a maximum of two hours twice a day, between 08.00-18.00
- The volunteer will be escorted by a member of the study team when walking through non-designated areas of the NIHR-CRF
- The volunteer must not have contact with immunosuppressed individuals
- The volunteer must not have any direct contact that could involve transfer of respiratory secretions to anyone during the admission period
- The volunteer must not use the main entrance of the hospital or shops or cafes within the hospital building
- When outside of the NIHR-CRF the volunteer must be contactable by mobile phone at all times and must have study emergency phone number stored on their phone to contact the clinical study team if necessary
- The volunteer must be able to be return to the NIHR-CRF within 30 minutes.
- The volunteer may receive a maximum of two guests at a time between 8.00 and 22.00, who must wear masks covering nose and mouth while in close proximity to the volunteer and must adhere to strict infection control procedures.

Following discharge – challenge and contact volunteers:

For the first two weeks following discharge volunteers must avoid crowded social environments such as pubs and clubs.

For the remainder of the study period:

- Volunteers must not have any contact with high risk of transmission with any individuals other than their declared and consented bedroom contact/corresponding challenge volunteer – such contact includes:
    - Bed sharing
    - Intimate/sexual contact
    - Contact that may involve transfer of respiratory secretions e.g. kissing
    - Sharing cutlery or drinking vessels
- Volunteers must not engage in oral sex
- Volunteers must avoid contact with immunosuppressed individuals

For peer review only

SUPPLEMENTARY TABLE 3 – STUDY TIMETABLE FOR CHALLENGE VOLUNTEERS

	Screening	Pre challenge	Admission					Follow up							Potential additional visits		
Timeline (days)	≤ 90	-5	0	1	2	3	4	7	10	14	28	56	90	92	Additional shedding check[b]	Triggered eradication[c]	Post triggered eradication check
Day		W	M	Tu	W	Th	F	M	Th	M	M	M					
Visit window		+/-2	0	0	0	0	0	+/-1	+/-1	+/-2	+/-3	+/-5	+/-7	-1 to 0[a]	0[d]	0[d]	0[a]
TOPS confirmation	+																
Volunteer Information Sheet	+																
Informed consent	+																
Infection control training	+						+										
Vital signs	+	(+)	+	+	+	+	+	(+)	(+)	(+)	(+)	(+)	(+)	(+)	(+)	(+)	(+)
Medical history	+																
Physical examination	+	(+)	(+)	(+)	(+)	(+)	(+)	(+)	(+)	(+)	(+)	(+)	(+)	(+)	(+)	(+)	(+)
Pregnancy test (females	+		+										+			+	
Urinalysis	+																
Electrocardiogram	+																
Review eligibility		+	+														
Inoculation			+														
Eradication													+			+	
Review of adverse events and concomitant medications		+	+	+	+	+	+	+	+	+	+	+	+	+	+	+	+
Throat swab 1	+	+	+		+	+	+	+	+	+	+	+	+	+	+	+	+
Nasal wash		+				+				+	+	+	+			+	
Throat swab 2 (microbiome)			+			+		+	+	+	+	+	+			+	
Nasosorption test			+			+				+	+	+	+			+	
Saliva sample			+			+				+	+	+	+			+	
Environmental samples				+	+	+	+	+	+	+	+	+	+		+	+	
Safety bloods	8		8			8		8		8	8	8	8			8	
Immunological blood tests			70					70		70	70	70	70			70	
Cumulative blood volume	8		86			94		172		250	328	406	484				

(+) If clinically indicated, [a]1-2 days after eradication, [b]If increased shedding seen at one timepoint from Day 14, [c]If early eradication triggered (see section 9.5.3), [d]As soon as possible after triggering results are known.

For peer review only

For peer review only - http://bmjopen.bmj.com/site/about/guidelines.xhtml

SUPPLEMENTARY TABLE 4 – STUDY TIMETABLE FOR CONTACT VOLUNTEERS

	**Screening**	**Challenge volunteer discharge**	**Follow up**					**Potential additional visits**	
Timeline (days)	≤ 90	4	14	28	56	90	92	Early / triggered eradication[c]	Early / triggered eradication check
Day		F	M	M	M	+/-7[a]	-1 to 0[c]	0[d]	-1 to 0[b]
Visit window		0	+/-2	+/-3	+/-5				
TOPS confirmation	+								
Volunteer Information Sheet	+								
Informed consent	+								
Reconfirm eligibility		+							
Infection control training	+	+							
Vital signs	+		(+)	(+)	(+)	(+)	(+)	(+)	(+)
Medical history	+								
Physical examination	+		(+)	(+)	(+)	(+)	(+)	(+)	(+)
Pregnancy test (females only)	+	+				+		+	
Urinalysis	+								
Electrocardiogram	+								
Eradication						+		+	
Review of adverse events and concomitant medications			+	+	+	+	+	+	+
Throat swab	+	+	+	+	+	+	+	+	+

(+) If clinically indicated, [a]Same day as corresponding challenge volunteer, [b]1-2 days after eradication, [c]If early eradication triggered, [d]As soon as possible after triggering results are known

For peer review only

BMJ Open

Protocol for a controlled human infection with genetically modified Neisseria lactamica expressing the meningococcal vaccine antigen NadA: A potent new technique for experimental medicine

Journal:	*BMJ Open*
Manuscript ID	bmjopen-2018-026544.R1
Article Type:	Protocol
Date Submitted by the Author:	18-Jan-2019
Complete List of Authors:	Gbesemete, Diane; University Hospital Southampton NHS Foundation Trust, NIHR Clinical Research Facility; University of Southampton , Faculty of Medicine<br>Laver, Jay; University of Southampton<br>de Graaf, Hans; University Hospital Southampton NHS Foundation Trust, NIHR Clinical Research Facility; University of Southampton , Faculty of Medicine<br>Ibrahim, Muktar; University of Southampton, Clinial & Experimental Sciences<br>Vaughan, Andrew; University of Southampton, Clinical & Experimental Sciences<br>Faust, Saul; University of Southampton, UK, NIHR Wellcome Trust Clincial Research Facility<br>Gorringe, Andrew; Public Health England Porton, Research<br>Read, Robert; University of Southampton; NIHR Southampton Biomedical Research Centre
**Primary Subject Heading**:	Infectious diseases
Secondary Subject Heading:	Immunology (including allergy)
Keywords:	INFECTIOUS DISEASES, meningitis, Neisseria meningitidis, genetically modified organisms, controlled human infection model

SCHOLARONE™
Manuscripts

Protocol for a controlled human infection with genetically modified *Neisseria lactamica* expressing the meningococcal vaccine antigen NadA: A potent new technique for experimental medicine

Authors:

Diane Gbesemete[1,2], Jay R Laver[3], Hans de Graaf[1,2], Muktar Ibrahim[1], Andrew Vaughan[2,3], Saul Faust[2,3], Andrew Gorringe[4], Robert C Read[1,3]

1. Faculty of Medicine, University Hospital Southampton NHS Foundation Trust, Southampton, UK
2. NIHR Clinical Research Facility, University Hospital Southampton NHS Foundation Trust, Southampton, UK
3. NIHR Southampton Biomedical Research Centre, University of Southampton
4. Pathogen Immunology Group, Public Health England, Salisbury, Wiltshire, UK

Corresponding author:

Diane Gbesemete, NIHR Clinical Research Facility, University Hospital Southampton NHS Foundation Trust, Southampton, SO30 3RE, UK

d.gbesemete@soton.ac.uk, 023 8120 4989

Key words:

Human challenge study, colonisation, *Neisseria lactamica*, Genetically modified organism

Word count:

5250

For peer review only

ABSTRACT

Introduction

Neisseria lactamica is a commensal organism found in the human nasopharynx and is closely related to the pathogen *Neisseria meningitidis* (meningococcus). Carriage of *N. lactamica* is associated with reduced meningococcal carriage and disease. We summarise an ethically approved protocol for an experimental human challenge study using a genetically modified strain of *N. lactamica* that expresses the meningococcal antigen NadA. We aim to develop a model to study the role of specific bacterial antigens in nasopharyngeal carriage and immunity, to evaluate vaccines for their efficacy in preventing colonisation, and to provide a proof of principle for the development of bacterial medicines.

Methods and analysis

Healthy adult volunteers aged 18-45 years will receive an intranasal inoculation of either the NadA containing strain of *N. lactamica* or a genetically modified, but wild type-equivalent control strain. These challenge volunteers will be admitted for 4.5 days observation following inoculation and will then be discharged with strict infection control rules. Bedroom contacts of the challenge volunteers will also be enrolled as contact volunteers. Safety, colonisation, shedding, transmission and immunogenicity will be assessed over 90 days after which carriage will be terminated with antibiotic eradication therapy.

Ethics and dissemination

This study has been approved by the Department for Environment, Food and Rural Affairs (DEFRA) [1] and South Central Oxford A Research Ethics Committee reference: 18/SC/0133. Findings will be published in peer-reviewed open access journals as soon as possible.

For peer review only

STRENGTHS AND LIMITATIONS OF THIS STUDY

- This human challenge study using a genetically modified organism will provide insight into the role of a specific bacterial antigen in nasopharyngeal carriage and immunity, and provide a novel means to test the herd-immunity potential of vaccines
- Safety is the first priority and has been considered at all points of the study design with extensive pre-clinical testing, a period of admission for close observation following inoculation and stringent infection control rules throughout the study
- The use of environmental sampling and regular contact volunteer sampling will provide new information regarding the shedding and transmission of respiratory tract organisms
- The planned inoculum dose is based on previous studies with wild type *N. lactamica* and may not be the optimal dose to achieve colonisation with the genetically modified strains
- The low number of participants may be insufficient to prove an effect of the expression of NadA on colonisation so further research may be required

For peer review only

INTRODUCTION

A controlled human infection experiment with a genetically modified *Neisseria lactamica* strain is currently underway. In the protocol, presented here, organisms are inoculated into the nasopharynx of healthy volunteers to study the immune response to the modified organisms expressing the gene of interest. Volunteers,colonised with the strain harboured in the nasopharynx,will be allowed to leave the clinical research facility after a 5 day period of observation. This implies deliberate release of a genetically modified organism so the protocol has been reviewed and approved by the United Kingdom Department for the Environment, Food and Rural Affairs (DEFRA)[1].

Neisseria lactamica and *Neisseria meningitidis* are Gram negative diplococci which both colonise the human nasopharynx. *Neisseria lactamica* is non-pathogenic, non-encapsulated and lactose fermenting and is a common commensal, particularly in young children [2 3]. In contrast *N. meningitidis* expresses polysaccharide capsule and although it usually colonises asymptomatically, it can in a minority of colonised individuals, cause invasive disease [4 5]. Due to recombination events, the organism exists in multiple clonal forms, with specific clonal complexes being characteristically associated with invasive disease[6]. Invasive meningococcal disease remains a significant global cause of morbidity and mortality with sporadic disease and small outbreaks throughout the world and significant epidemics occurring in the meningococcal belt of sub-Saharan Africa[7].

Carriage of *N. lactamica* and *N. meningitidis*

Of note, *N. lactamica* appears to provide commensal-related protection against meningococcal disease. Age-specific rates of *N. meningitidis* carriage and disease are inversely proportional to carriage of *N. lactamica* [8-10]. The highest rate of natural carriage of *N. lactamica* occurs in infants. This then wanes in toddlers and older children and by adolescence carriage is approximately 1% [2 8]. Carriage of *N. meningitidis* is low in infants, increasing gradually throughout childhood and peaking

in adolescence with the highest rates of carriage seen in teenagers and University students [11].

The mechanism of this epidemiological relationship is as yet undetermined. It is probably not due to cross-protective antibody production; the early years of life associated with high rates of *N. lactamica* carriage predate the development of natural bactericidal meningococcal antibodies [4]. Other postulated mechanisms include microbial competition, innate immune responses triggered by *N. lactamica* colonisation and cross-reactive non-humoral acquired immunity [12 13].

Human challenge with *Neisseria lactamica*

A controlled human infection model of *N. lactamica* colonisation has been utilised to investigate the mechanism of this natural effect. Previous studies have shown that human challenge with wild type *N. lactamica* is safe and can induce long standing colonisation. Over 350 healthy adult volunteers have been experimentally nasally inoculated with wild type *N. lactamica* in previous studies. The colonisation fraction (the percentage of individuals who are colonised after challenge) was 35-65% [12 13]. Colonisation resulted in the development of humoral immunity to *N. lactamica* but no evidence of cross reactive bactericidal antibodies to *N. meningitidis*. Some cross-reactive opsonophagocytic antibody production occurred but was rather weak. [13]. In another large study, successful colonisation with *N. lactamica* was associated with the displacement of pre-existing meningococcal carriage, and inhibition of acquisition of *N. meningitidis* [12] supporting the role of *N. lactamica* carriage in protection from meningococcal carriage and therefore disease.

Meningococcal vaccines

Glycoconjugate vaccines directed against capsular antigens for serogroups C, A, W-135 and Y have been in use globally for several years. These have had dramatic effects on disease incidence, which is probably mostly due to herd protection conferred by vaccine-induced modification of colonisation reducing inter-host transmission [14 15]. Recent vaccine developments include a new subcapsular vaccine, 4CMenB (Bexsero), which induces bactericidal antibodies against a range of strains, including serogroup B, and protects vaccinated infants against disease [16]. In view of

For peer review only

the importance of carriage-reduction for herd immunity, a large prospective randomised study was done to measure this, but the effect of Bexsero on carriage of *N. meningitidis* was found to be relatively modest and delayed until 3 months after vaccination [17], with no evidence of an effect on carriage of the serogroup B organisms carried by the participants.

More rapidly effective and longer lasting vaccines are required, particularly to halt transmission during epidemics in the meningitis belt of sub-Saharan Africa. Successful future vaccines should maximise herd immunity by targeting carriage and transmission. The development of such vaccines requires a greater understanding of mucosal immune mechanisms and the specific antigens involved in colonisation.

The meningococcal antigen NadA

In this human challenge study volunteers will receive intranasal inoculation with a genetically modified (GM) strain of *N. lactamica* expressing the meningococcal antigen NadA. This antigen is being used because it is well defined, and one of the 4 strongly immunogenic components of the Bexsero vaccine. Bexsero and has been demonstrated to be immunogenic in terms of generating serum bactericidal antibodies against *N. meningitidis* strains that express NadA [18] and moderately effective in reducing acquisition of nasopharyngeal carriage of *N. meningitidis* over the course of 12 months after vaccination [17]. NadA expression by *N. lactamica* may induce systemic and mucosal immunity to NadA. When studied alongside a control strain, use of a GMO *N. lactamica* expressing NadA could permit advanced study of the mechanisms underlying mucosal immunity and carriage-reduction. Furthermore, a GMO *N. lactamica* expressing NadA might exhibit enhanced protection against carriage of virulent *N. meningitidis*.

Rationale for this study

The rationale for this study is to pilot the use of the transformed commensal *N. lactamica* as an experimental medicine tool to study immunity to meningococcal antigens in humans, and to investigate the potential utility of genetically transformed commensals as tools to investigate the efficacy of vaccines to prevent colonisation of organisms expressing specific antigens. Finally, expression of NadA might lead to

For peer review only

increased efficiency of harmless colonisation by *N. lactamica* and prompt the development of this GMO as a bacterial medicine.

For peer review only

METHODS AND ANALYSIS

Study overview

This is a prospective controlled human challenge study in which challenge volunteers will be inoculated intranasally with *Neisseria lactamica* (recipient strain Y92-1009) genetically modified to express NadA (the intervention strain) or a control genetically modified strain. An inoculum dose of 10^5 CFU will be used for both strains. Following inoculation, challenge volunteers will be admitted to Southampton National Institute for Health Research Clinical Research Facility (NIHR CRF) for 4.5 days. A further group of volunteers, who are close contacts of the participants will be enrolled to detect transmission of the inoculated strains. Safety parameters, colonisation, shedding, transmission and immunogenicity will be assessed during the admission period and over a follow up period of approximately 3 months. Colonisation will be terminated with antibiotic eradication therapy on Day 90, for all challenge and contact volunteers. The planned study period is from May 2018 to May 2020.

Study objectives

The objectives of this study are to establish the safety and NadA-specific immunogenicity of nasal inoculation with the intervention strain of GM *N. lactamica* and to assess subsequent shedding and transmission. A further objective is to assess the efficacy of ciprofloxacin eradication therapy. These objectives and the study endpoints are summarised in Table 1 below:

For peer review only

	Objectives	Endpoints
Co-primary objectives	To establish the safety of nasal inoculation of healthy volunteers with a genetically modified strain of *Neisseria lactamica* expressing NadA	Occurrence of unsolicited adverse events within the study period
		Occurrence of serious adverse events within the study period
	To assess the NadA specific immunity in healthy volunteers following nasal inoculation with *Neisseria lactamica* expressing NadA	Rise in serological specific IgG titre (anti-NadA) comparing day 0 versus days 14 to 90 comparing volunteers colonised by one of the two GMOs
		Rise in mucosal specific antibody titre comparing day -5 versus days 3 to 90 and comparing volunteers colonised with the two GMOs
		Change in nasal cytokine profile comparing day 0 versus days 3 to 90 and comparing volunteers colonised with the two GMOs
Secondary objectives	To assess the shedding of genetically modified *Neisseria lactamica* following nasal inoculation	Culture of GM *N. lactamica* from environmental samples – comparing intervention and control groups
	To assess the transmission of genetically modified *Neisseria lactamica* to bedroom contacts of inoculated volunteers	Culture of GM *N. lactamica* from throat swabs taken from contact volunteers from day 4 until day 90 – comparing intervention and control groups
	To assess the efficacy of a single dose of Ciprofloxacin in eradicating carriage of genetically modified *Neisseria lactamica*	Culture of GM *N. lactamica* from throat swabs taken at the eradication visit in comparison to post-eradication visit in challenge and contact volunteers

Table 1 – Objectives and Endpoints

For peer review only

Genetically modified *Neisseria lactamica*

The intervention strain

The intervention strain (*Neisseria lactamica* strain Y92-1009), has been transformed by the integration of the *N. meningitidis* gene *nadA* (NEIS1969)*,* leading to expression of NadA. The NadA protein is a member of the type V autotransporter family of outer membrane proteins, and in *N. meningitidis* is associated with an increased level of adhesion to and invasion of human epithelial cell lines. The inserted gene is derived from *Neisseria meningitidis* strain MC58, which contains *nadA* allele 1.The presence of the *nadA* gene in the genome is associated with hypervirulent lineages of *N. meningitidis*, but NadA surface expression has not been shown to be causal for increased virulence. Detailed molecular microbiological information can be found within the published DEFRA approval notice. [1]

The control strain

The control strain has been genetically modified in exactly the same way as the intervention strain, except that it does not contain the coding sequence for the *nadA* gene. In terms of gene content and behaviour in the laboratory, this strain is extremely similar to wild type. Using this strain as a control inoculum is superior to using the wild type strain as the changes made to the genetic architecture and gene regulation are identical to the intervention strain apart from the insertion of *nadA*.

Pre-clinical safety data

Both strains have been demonstrated to remain acutely susceptible to killing by normal human serum and retain sensitivity to the antibiotics used clinically to treat meningococcal disease (rifampicin, ciprofloxacin and ceftriaxone). Pre-clinical testing [1] has shown that the NadA autotransporter is functionally expressed in the intervention strain, the NadA protein is strongly immunogenic in the context of expression in *N. lactamica* and that expression of NadA does not significantly increase pathogenicity of the commensal in a murine model of infection. Neither strain has an increased propensity to become transformed by exogenous sources of DNA, which might otherwise allow it to acquire virulence factors such as an extracellular capsule, as compared to the wild type strain.

For peer review only - http://bmjopen.bmj.com/site/about/guidelines.xhtml

Quality assessment and control

Preparation, storage and monitoring of the challenge strains will be carried out to GMP-like standards at the University of Southampton. The dose and purity of the inoculum will be determined after inoculation for quality assessment.

The inoculum dose

Based on the previous *N. lactamica* human challenge studies it is estimated that 50% of volunteers will be colonised 1-2 weeks after inoculation at this dose[13]. Fifty per cent (50%) has been chosen as an acceptable colonisation rate because it is below a "saturating" dose and therefore avoids the difficulties of interpretation of a challenge dose that is much higher than physiologically appropriate.

Study volunteers

Challenge volunteers

Healthy volunteers aged 18-45 years will be recruited and challenged until 11 volunteers in each group are colonised with GM *N. lactamica* at day 14 or up to a maximum of 22 inoculated volunteers in each group.

Contact volunteers

Contact volunteers are bedroom contacts of challenge volunteers, defined as individuals who share a bedroom on at least one occasion during the study period. A maximum of one contact volunteer may be recruited per challenge volunteer and contact volunteers must give informed consent prior to inoculation of the corresponding challenge volunteer. Bedroom contacts who are under 18 or who are immunocompromised will be excluded from participation, as will their corresponding challenge volunteer.

Eligibility criteria

We will not recruit from vulnerable groups such as those with impaired capacity. Those with close contact with potentially vulnerable people such as small children

For peer review only

and immunocompromised individuals will be excluded. Specific inclusion and exclusion criteria can be found in Supplementary table 1.

Infection control agreement

Both challenge and contact volunteers must provide written infection control agreement prior to enrolment, which will include agreement to have no other bedroom contacts during the study period. Details of the infection control requirements can be found in Supplementary table 2.

Study setting

The challenge procedure, admission and follow up visits will take place in the NIHR CRF at University Hospital Southampton NHS Foundation Trust.

Recruitment

Participants will be recruited via a variety of media including ethically approved adverts displayed within the hospital, on Southampton NIHR CRF websites, social media and circulated literature, the Southampton CRF database of healthy volunteers, presentations and press releases. Individuals who express an interest will be sent a volunteer information sheet. Volunteers will be offered reimbursement for their time, travel and inconvenience.

Study timeline

Challenge and contact volunteers will be enrolled from the date of screening, up to 90 days prior to the challenge procedure, until day 92 post challenge. The duration of volunteer participation will therefore be up to approximately 6 months. An overview of the study timeline is shown in Figure 1 below. Details of study procedures are shown in Supplementary table 3 (Challenge volunteers) and Supplementary table 4 (Contact volunteers).

Figure 1: Study timeline

For peer review only

Screening

Potential challenge and contact volunteers will be invited to separate screening visits up to 90 days prior to challenge. At these screening visits they will be fully informed of all aspects of their involvement in the study, be given an opportunity to ask questions, to give informed consent and to undergo a medical screening to determine eligibility. Challenge volunteers will be asked to complete a pre-consent questionnaire to ensure their understanding of the study and their medical history will be confirmed with their GP. The infection control guidelines (see Supplementary table 2) will be explained to all volunteers and they will be asked to complete a questionnaire to confirm their understanding of these guidelines, and to sign an agreement to follow these guidelines throughout the study period. Challenge volunteers will attend a pre-challenge visit the week prior to their challenge to ensure that they remain eligible.

First volunteers

For each GM strain the first volunteers will be challenged individually and then in pairs with a safety review after volunteers 1, 3 and 5. Further volunteers will be challenged in groups of a maximum of 5.

Challenge

Challenge volunteers will be admitted to a designated area of the NIHR CRF on the morning of their challenge procedure. Ongoing informed consent and eligibility will be confirmed and clinical samples will be taken for baseline immunology.

The inoculum will be prepared from frozen stocks and will be administered by a study doctor following study-specific standard operating procedures. The challenge will take place in an environmental chamber within the CRF. The challenge volunteer will be positioned supine with neck extended and breathing normally through their mouth. 0.5ml of inoculum will be administered slowly from a pipette into each nostril. The residual inoculum will be analysed to confirm the administered dose and purity. Public Health Southampton will be informed of all participants who have been challenged with the GMOs.

For peer review only

Admission

During admission, challenge volunteers will have access to an individual bedroom, shared bathroom facilities and a shared recreational area. Clinical observations and any symptoms will be recorded approximately every 4 hours and a study doctor will review volunteers twice a day. Clinical and environmental samples will be taken as detailed in table 2 below to assess safety, colonisation, immunogenicity and shedding.

	Day 0	Day 1	Day 2	Day 3	Day 4
Vital signs	Pre inoculation then 4 hourly	4 hourly	4 hourly	4 hourly	4 hourly
Review of adverse events	4 hourly	4 hourly	4 hourly	4 hourly	4 hourly
Medical review	x 2	x 2	x 2	x 2	x 2
Pregnancy test (females only)	+				
Review eligibility	+				
Inoculation	+				
Throat swab (culture)	+		+	+	+
Throat swab (microbiome)	+			+	
Nasal wash				+	
Nasosorption test	+			+	
Saliva sample	+			+	
Environmental samples		+	+	+	+
Safety bloods (ml)	8			8	
Immunological blood tests (ml)	70				

Table 2 – Study procedures during admission

Prior to discharge of the challenge volunteer, the contact volunteer will attend to confirm ongoing consent and eligibility and the infection control procedures will be reiterated to both challenge and contact volunteers.

Follow up

Following challenge volunteer discharge, volunteers will be monitored for adverse events, colonisation, shedding, transmission and immunogenicity as detailed in in

For peer review only

Supplementary table 3 (Challenge volunteers) and Supplementary table 4 (Contact volunteers).

Adverse events

Adverse events will be monitored at each follow up visit. In addition to this volunteers will be encouraged to contact the study team at any point during the study in the event any symptoms develop.

Colonisation

Colonisation will be assessed by culture of throat swabs and nasal washes. Colonisation density will be estimated by qPCR and comparison will be made between the intervention and control groups.

Shedding

Shedding of GM *N. lactamica* from inoculated challenge volunteers will be assessed by microbiological analysis of environmental samples. Comparison of shedding will be made between the intervention and control challenge volunteers. Environmental sampling will include culture and PCR of face mask samples and air samples taken within an environmental chamber during aerosol producing activities.

A challenge volunteer in the intervention group will be considered to have increased shedding at a particular time point if they have a 10-fold increase in shedding in comparison to the average shedding seen at the same time point in colonised control group volunteers to date. This is a nominal figure agreed with the statutory authority (UK Department for the Environment, Food and Rural Affairs) because of the unpredictable scale and frequency of this event which will not permit a prospective, statistically-based assessment of potentially hazardous release to the environment. If increased shedding is seen at any point from the Day 14 visit then the volunteer will be asked to attend as soon as possible for an additional shedding check visit. If increased shedding is seen at two consecutive visits this will be considered enhanced shedding.

For peer review only

Transmission

Transmission will be assessed by culture and PCR of throat swabs from contact volunteers. Comparison will be made between the intervention and control groups.

Immunogenicity

Mucosal and systemic immunogenicity will be investigated. Saliva and nasal secretions will be collected for assessment of mucosal immunogenicity and blood samples for systemic humoral and cellular responses.

Eradication

For peer review only

Antibiotic eradication therapy will be given to all challenge and contact volunteers with a throat swab to confirm successful eradication after a maximum of 48 hours. Standard eradication will be given to all volunteers at Day 90 (regardless of colonisation status) with a confirmatory throat swab on Day 92. Eradication therapy may be given at an earlier time point under specific circumstances.

Triggered eradication may be given to volunteers at any time point due to:

- Safety concerns in the challenge volunteer or corresponding contact volunteer, at the discretion of the study team
- Enhanced shedding from the challenge volunteer
- Study withdrawal for any other reason

If eradication is triggered for a challenge or contact volunteer then their corresponding challenge or contact volunteer (if applicable) will receive eradication therapy on the same day and both volunteers will be withdrawn from the study.

In addition to this, contact volunteers found to be colonised with GM *N. lactamica* at any point may receive early eradication therapy, as ongoing colonisation of contact volunteers is not required to fulfil the study objectives. In this case the corresponding challenge volunteer will not receive eradication therapy and both will continue in the study as planned.

A single dose of 500 mg ciprofloxacin will be taken under supervision of the study team. All female volunteers will have a pregnancy test prior to eradication. In the

event of a positive pregnancy test, alternative eradication therapy will be used – Rifampicin 600 mg bd for 48 hours.

Both Rifampicin and Ciprofloxacin, as oral antibiotics, have been shown to be effective in eradicating carriage of *N. meningitidis* [19], and are regularly used as post exposure prophylaxis [20]. Both GM strains are also sensitive to these antibiotics.

Study holding rules

An independent external safety committee will review the safety aspects of the study on a regular basis and in the event of any significant safety concerns. Colonisation, shedding, transmission and clinical parameters will be closely monitored throughout the study. In the event of a study holding criterion being met the study will be paused for a safety review. No further volunteers will be challenged until the data have been reviewed by the external safety committee and study continuation approved.

Enhanced colonisation

The expression of NadA by the intervention strain of GM *N. lactamica* is expected to be associated with either an increase or a decrease in colonisation frequency or density compared to wild type. Colonisation rate and density estimation will be monitored but an increase in colonisation alone will not trigger a study pause unless associated with sustained enhanced shedding, transmission or safety concerns.

Enhanced shedding

Enhanced shedding triggering early eradication in 3 or more of the first 5 volunteers to receive the intervention strain or in >50% of ongoing challenge volunteers in the intervention group will trigger a study pause.

Enhanced transmission

Transmission of either strain of GM *N. lactamica* to 3 of the first 5 or >50% of ongoing contact volunteers will trigger a study pause.

GM N. lactamica disease

If antibiotic treatment (IV ceftriaxone or IV chloramphenicol) is given to any volunteer due to possible GM *N. lactamica* disease then a study pause will be triggered.

For peer review only

Sample size

We are aiming to achieve colonisation in 10 challenge volunteers for each of the GM strains. This is based on a previous experimental *N. lactamica* challenge study, which showed a significant rise in serological antibody titre against *N. lactamica* over 2 weeks [13]. This gave SDs on a log-10 scale of 0.11 for IgA saliva and 0.26 for serum total IgG. For this study, using the SD of 0.26 we will be able to confirm a 4 fold rise of anti-NadA with 10 carriers of *N. lactamica* expressing NadA with 90% power using analysis of variance.

Allowing for a drop out rate of approximately 10%, we will therefore recruit challenge volunteers until we have 11 individuals colonised for each group up to a maximum of 22 volunteers for each group. Estimating a colonisation fraction of 50%, approximately 44 individuals will be enrolled as challenge volunteers. A maximum of one contact volunteer will be enrolled per challenge volunteer.

Patient and Public Involvement

A PPI group was consulted during the early stages of study design to discuss the implications of human challenge with a genetically modified organism. An important suggestion arising from this consultation was to seek information about the potential for spread of infection which we have discussed further with PHE experts and DEFRA. As a result of these discussions, our protocol includes close monitoring of environmental shedding and transmission to sleeping partners with specific action points in the event that there is evidence of enhanced shedding into the environment. Suggestions from the PPI consultation were also used in the design of the volunteer information sheet.
In addition, formal and informal feedback from volunteers involved in other human challenge trials in the NIHR Clinical Research Facility Southampton has been used to refine the design of this study and preparation of the admission area.

Participants in this study will be provided with a lay summary of the results once available.

For peer review only

ETHICS AND DISSEMINATION

As this study involves the deliberate release of genetically modified bacteria into the community it has been considered and approved by the responsible government ministry - the Department for Environment, Food and Rural Affairs [1].

It has also been reviewed and approved by South Central Oxford A Research Ethics Committee (SC/18/0113) and by the UK Health Research Authority (IRAS ID 235090). Results will be published in peer-reviewed journals once available.

For peer review only

DISCUSSION

Human challenge with a genetically modified organism – safety considerations

This study will result in the deliberate release of two genetically modified organisms (GMOs). One previous study has been published in which volunteers were deliberately inoculated with a GMO that has therefore potentially been released into the general population. In that study, carried out in Sweden, a genetically modified attenuated *Bordetella pertussis* strain was constructed as a vaccine candidate. This was administered nasally, in order to mimic natural infection without inducing disease and volunteers were subsequently followed up as outpatients[21].

In the United Kingdom the deliberate release of a GMO requires DEFRA approval. This protocol has therefore been reviewed by DEFRA who have considered the potential for colonisation of other members of the general population, and have given approval of the study.

During the design of this study, our priority has been to ensure the safety of the volunteers to limit the potential for transmission to close contacts of the volunteers, study team members and the wider population. A number of safety considerations have been incorporated into the protocol and an independent external safety committee will review the safety aspects of the study on a regular basis.

For peer review only

Safety of GM *N. lactamica*

N. lactamica is a non-virulent commensal organism and there have been no safety concerns in previous challenge studies with the wild type organism. There is no evidence to suggest that the genetically modified strains will be more likely than wild type to cause invasive disease, as the organisms are non-capsulate and highly susceptible to killing by human serum. Pre-clinical work has indicated that the GMOs are stable, do not undergo recombination events at higher frequency than wild type

and are non-virulent when inoculated into mice. We therefore consider that the likelihood of the GMO causing any disease is extremely low.

Safety of challenge and contact volunteers

For each strain, the first five challenges will be staggered with a safety review between challenges. All challenged volunteers will be admitted to Southampton NIHR CRF for close observation for 4.5 days following challenge. The period of risk of development of invasive meningococcal disease is the first 48 hours following acquisition so in the unlikely event of any volunteer developing symptoms it would be expected to occur within this period of admission. The NIHR CRF is funded and staffed to allow the delivery of higher risk experimental studies and is located within an NHS hospital so study nurses will be immediately available, study doctors will be contactable and able to attend and full NHS clinical services will be present within the same building if required. Following discharge all volunteers will be monitored regularly for adverse events and will be given a 24 hour phone number to contact the study team.

Minimising onward transmission

Transmission occurs through close contact and previous studies looking at the transmission of *N. lactamica* and *N. meningitidis* suggest that household members, and in particular bedroom-sharers of colonised individuals are those at highest risk of acquisition of carriage [22-24]. Bedroom sharers of challenge volunteers are therefore the most relevant community members to screen for transmission and so will give informed consent and will be enrolled as contact volunteers for this purpose. Potential challenge or contact volunteers with household members or other close contacts who may be at increased risk of acquisition of carriage or of *N. lactamica* disease will be excluded from the study.

Other infection control measures include the use of PPE, strict infection control guidelines, and close monitoring of shedding and transmission. These measures have been designed to limit the potential onward transmission of the inoculated bacteria to study team members, vulnerable individuals and to the general

For peer review only

population. In addition all volunteers will receive eradication therapy prior to study completion, regardless of their colonisation status.

The benefit of a human challenge model

A greater understanding of the mucosal immune mechanisms of protection from colonisation is essential for the development and evaluation of new vaccines, specifically ones targeting colonisation and transmission. The most direct and effective way to achieve this is experimental controlled human infection. This model can be used to investigate in detail components of mucosal and systemic immunity activated in real time following infection with a defined antigen. Also, this model could be used to investigate vaccine efficacy. For example, healthy volunteers who have received a study vaccine could then be challenged with a defined organism expressing constituent antigens. Monitoring carriage of the challenge bacterium over time would then provide information of the efficacy of the vaccine in the prevention of colonisation. Experimental human challenge with pathogens of interest such as *N. meningitidis* would be potentially hazardous and therefore raise significant ethical and logistical issues. The use of a harmless commensal organism that has been transformed to express specific antigens could be a safe and effective alternative.

N. lactamica is an appropriate organism to be transformed for this purpose. It is a well-studied and characterised commensal organism, which is known to exclusively colonise the human nasopharynx. It is genetically very similar to *N. meningitidis*, sharing approximately 67% of the genes believed to be associated with meningococcal virulence [25]. Despite this, *N. lactamica* is known to be non-virulent and has been used safely in previous human challenge studies.

N. lactamica is the only member of the genus *Neisseria* which is able to ferment lactose due to the activity of β-D-galactosidase coded for by the gene *lacZ.* This causes colonies to grow blue on the chromogenic substrate 5-bromo-4-chloro-3-indolyl β-D-galactopyranoside (X-gal). This characteristic has been utilised in our study; both of our GM strains have been derived from a *lacZ* deficient strain of *N. lactamica* Y92-1009 (Δ*lacZ*), which grows as white colonies on X-gal-containing medium. During the transformation process *lacZ* has been re-integrated as a marker

For peer review only

of successful transformation, thus allowing screening for successful transformants on the basis of blue/white colony formation on X-gal-containing medium. This has been done to completely avoid the use of genes coding for resistance to antibiotics and to eliminate the risk of our challenge experiment disseminating antimicrobial resistance genes into the nasopharyngeal microbiome.

The meningococcal antigen NadA has been chosen as the specific antigen for this study. NadA is a component of the Bexsero vaccine and is known to be potently immunogenic so successful colonisation is likely to induce the production of specific anti-NadA antibodies. Indeed, in a murine nasal challenge model, wherein genetically modified *Streptococcus gordonii* expressing meningococcal NadA was used to inoculate mice, colonised subjects produced systemic anti-NadA bactericidal antibodies and localised anti-NadA IgA [26]. The *nadA* gene is associated with hypervirulent strains of *N. meningitidis* and was present in 50% of strains isolated from cases of meningococcal disease [27]. NadA has a role in increased adhesion and invasion into human epithelial cells [28] so NadA expression may therefore increase the ability of *N. lactamica* to colonise the nasopharynx. However *nadA* is absent from some virulent strains and the majority of non-virulent strains of *N. meningitidis*, which may limit the potential for cross-reactive immunity [27 29]. In addition, as NadA is so potently immunogenic, expression may in fact reduce the duration of colonisation due to enhanced clearance.

Once this human challenge model has been shown to be safe and effective it could potentially be used to study other meningococcal antigens, or indeed antigens from other respiratory mucosal pathogens.

The potential for use as a bacterial medicine

Carriage of wild type *N. lactamica* appears to be protective against meningococcal disease, at least partly due to physical competition. The modification of *N. lactamica* to express an adhesin such as NadA could plausibly improve the colonisation fraction or colonisation duration.

For peer review only

Colonisation with *N. lactamica* has been shown to result in some cross-reactive acquired immunity to *N. meningitidis*, but this is insufficient to be fully protective [13]. Genetic modification of *N. lactamica* to express a meningococcal antigen known to be potently immunogenic may lead to the production of anti-meningococcal serum bactericidal antibodies (SBA).

If successful, these improvements in the protective effect of induced colonisation with *N. lactamica* may lead to its potential use as a bacterial medicine.

Conclusion

The successful and safe colonisation of healthy volunteers with genetically modified strains of *N. lactamica* will pave the way for further challenge studies involving transformants which express other meningococcal antigens, and potentially antigens expressed by other pathogens. These challenge models will lead to a greater understanding of mucosal immune responses to colonisation and infection, provide a platform for the development and assessment of improved vaccines, and may lead to the development of novel bacterial medicines.

AUTHORS' CONTRIBUTIONS

The study was designed by DG, JRL and RCR with contributions from the other authors, HdG, MI, AV, SF, and AG. The first drafts of this manuscript were prepared by DG, JRL and RCR and then HdG, MI, AV, SF, and AG contributed to editing and approval of the final version of this manuscript.

ACKNOWLEDGEMENTS

We would like to acknowledge the input of a Public and Patient Involvement group in the early design stage of this study.

For peer review only

FUNDING STATEMENT

This work will be supported by the Medical Research Council (Grant MR/N026993/1) “Pathfinder: Experimental Human Challenge with Genetically Modified Commensals to Investigate Respiratory Tract Mucosal Immunity and Colonisation” and the MRC Confidence in Concept Award, with additional funding from Experimental Medicine by the National Institute for Health Research through support from the Southampton NIHR CRF and the Biomedical Research Centre. The development of the technology underpinning the genetic modification was funded by the Medical Research Council (A genetically modified nasopharyngeal commensal as a platform for bacterial therapy, MR/N013204/1).

COMPETING INTERESTS STATEMENT

JRL and RR declare a potential conflict of interest: The patent WO2017103593-A1 `New modified *Neisseria lactamica* transformed with recombinant DNA encoding heterologous protein, used for e.g. prophylactic treatment of pathogenic infection, preferably meningococcal infection`, is assigned to the University of Southampton, with Dr JR Laver, and Professor RC Read as inventors.

For peer review only

REFERENCES

1. Laver JR, Read RC. Genetically Modified Organisms: University of Southampton (17/R50/01) 2017 [Available from: https://www.gov.uk/government/publications/genetically-modified-organisms-university-of-southampton-17r5001
2. Bennett JS, Griffiths DT, McCarthy ND, et al. Genetic diversity and carriage dynamics of Neisseria lactamica in infants. *Infection and immunity* 2005;73(4):2424-32. doi: 10.1128/IAI.73.4.2424-2432.2005
3. Liu G, Tang CM, Exley RM. Non-pathogenic Neisseria: members of an abundant, multi-habitat, diverse genus. *Microbiology (Reading, England)* 2015;161(7):1297-312. doi: 10.1099/mic.0.000086
4. Trotter CL, Gay NJ, Edmunds WJ. The natural history of meningococcal carriage and disease. *Epidemiol Infect* 2006;134(3):556-66. doi: 10.1017/S0950268805005339
5. Stephens DS, Greenwood B, Brandtzaeg P. Epidemic meningitis, meningococcaemia, and Neisseria meningitidis. *Lancet (London, England)* 2007;369(9580):2196-210. doi: 10.1016/S0140-6736(07)61016-2
6. Read RC. Neisseria meningitidis; clones, carriage, and disease. *Clinical microbiology and infection : the official publication of the European Society of Clinical Microbiology and Infectious Diseases* 2014;20(5):391-95. doi: 10.1111/1469-0691.12647
7. Harrison LH, Trotter CL, Ramsay ME. Global epidemiology of meningococcal disease. *Vaccine* 2009;27 Suppl 2:63. doi: 10.1016/j.vaccine.2009.04.063
8. Gold R, Goldschneider I, Lepow ML, et al. Carriage of Neisseria meningitidis and Neisseria lactamica in infants and children. *The Journal of infectious diseases* 1978;137(2):112-21.
9. Cartwright KA, Stuart JM, Jones DM, et al. The Stonehouse survey: nasopharyngeal carriage of meningococci and Neisseria lactamica. *Epidemiol Infect* 1987;99(3):591-601.
10. Olsen SF, Djurhuus B, Rasmussen K, et al. Pharyngeal carriage of Neisseria meningitidis and Neisseria lactamica in households with infants within areas with high and low incidences of meningococcal disease. *Epidemiology and infection* 1991;106(3):445-57.
11. Christensen H, May M, Bowen L, et al. Meningococcal carriage by age: a systematic review and meta-analysis. *The Lancet Infectious diseases* 2010;10(12):853-61. doi: 10.1016/S1473-3099(10)70251-6
12. Deasy AM, Guccione E, Dale AP, et al. Nasal Inoculation of the Commensal Neisseria lactamica Inhibits Carriage of Neisseria meningitidis by Young Adults: A Controlled Human Infection Study. *Clinical infectious diseases : an official publication of the Infectious Diseases Society of America* 2015;60(10):1512-20. doi: 10.1093/cid/civ098 [published Online First: 2015/03/31]
13. Evans CM, Pratt CB, Matheson M, et al. Nasopharyngeal colonization by Neisseria lactamica and induction of protective immunity against Neisseria meningitidis. *Clinical infectious diseases : an official publication of the*

For peer review only

Infectious Diseases Society of America 2011;52(1):70-7. doi: 10.1093/cid/ciq065 [published Online First: 2010/12/15]
14. Maiden MC, Ibarz-Pavón AB, Urwin R, et al. Impact of meningococcal serogroup C conjugate vaccines on carriage and herd immunity. *The Journal of infectious diseases* 2008;197(5):737-43. doi: 10.1086/527401
15. Trotter CL, Maiden MC. Meningococcal vaccines and herd immunity: lessons learned from serogroup C conjugate vaccination programs. *Expert review of vaccines* 2009;8(7):851-61. doi: 10.1586/erv.09.48
16. Parikh SR, Andrews NJ, Beebeejaun K, et al. Effectiveness and impact of a reduced infant schedule of 4CMenB vaccine against group B meningococcal disease in England: a national observational cohort study. *Lancet (London, England)* 2016;388(10061):2775-82. doi: 10.1016/S0140-6736(16)31921-3
17. Read RC, Baxter D, Chadwick DR, et al. Effect of a quadrivalent meningococcal ACWY glycoconjugate or a serogroup B meningococcal vaccine on meningococcal carriage: an observer-blind, phase 3 randomised clinical trial. *Lancet (London, England)* 2014;384(9960):2123-31. doi: 10.1016/S0140-6736(14)60842-4
18. Read RC, Dull P, Bai X, et al. A phase III observer-blind randomized, controlled study to evaluate the immune response and the correlation with nasopharyngeal carriage after immunization of university students with a quadrivalent meningococcal ACWY glycoconjugate or serogroup B meningococcal vaccine. *Vaccine* 2017;35(3):427-34. doi: 10.1016/j.vaccine.2016.11.071
19. Gaunt PN, Lambert BE. Single dose ciprofloxacin for the eradication of pharyngeal carriage of Neisseria meningitidis. *J Antimicrob Chemother* 1988;21(4):489-96.
20. Fraser A, Gafter-Gvili A, Paul M, et al. Antibiotics for preventing meningococcal infections. *The Cochrane database of systematic reviews* 2006(4):CD004785. doi: 10.1002/14651858.CD004785.pub3 [published Online First: 2006/10/21]
21. Thorstensson R, Trollfors B, Al-Tawil N, et al. A phase I clinical study of a live attenuated Bordetella pertussis vaccine--BPZE1; a single centre, double-blind, placebo-controlled, dose-escalating study of BPZE1 given intranasally to healthy adult male volunteers. *PloS one* 2014;9(1) doi: 10.1371/journal.pone.0083449
22. Simmons G, Martin D, Stewart J, et al. Carriage of N. lactamica in a population at high risk of meningococcal disease. *Epidemiology and infection* 2000;125(1):99-104.
23. Cartwright KA, Stuart JM, Robinson PM. Meningococcal carriage in close contacts of cases. *Epidemiology and infection* 1991;106(1):133-41.
24. Kristiansen BE, Tveten Y, Jenkins A. Which contacts of patients with meningococcal disease carry the pathogenic strain of Neisseria meningitidis? A population based study. *BMJ (Clinical research ed)* 1998;317(7159):621-25.
25. Snyder LA, Saunders NJ. The majority of genes in the pathogenic Neisseria species are present in non-pathogenic Neisseria lactamica, including those designated as 'virulence genes'. *BMC genomics* 2006;7:128. doi: 10.1186/1471-2164-7-128
26. Ciabattini A, Giomarelli B, Parigi R, et al. Intranasal immunization of mice with recombinant Streptococcus gordonii expressing NadA of Neisseria meningitidis induces systemic bactericidal antibodies and local IgA. *Vaccine* 2008;26(33):4244-50. doi: 10.1016/j.vaccine.2008.05.049

For peer review only

27. Comanducci M, Bambini S, Caugant DA, et al. NadA diversity and carriage in Neisseria meningitidis. *Infection and immunity* 2004;72(7):4217-23. doi: 10.1128/IAI.72.7.4217-4223.2004

28. Capecchi B, Adu-Bobie J, Di Marcello F, et al. Neisseria meningitidis NadA is a new invasin which promotes bacterial adhesion to and penetration into human epithelial cells. *Molecular microbiology* 2005;55(3):687-98. doi: 10.1111/j.1365-2958.2004.04423.x

29. Vogel U, Taha M-KK, Vazquez JA, et al. Predicted strain coverage of a meningococcal multicomponent vaccine (4CMenB) in Europe: a qualitative and quantitative assessment. *The Lancet Infectious diseases* 2013;13(5):416-25. doi: 10.1016/S1473-3099(13)70006-9

For peer review only

FIGURES:

Figure 1 – Study timeline

TABLES:

Table 1 – Objectives and endpoints

Table 2 – Study procedures during admission

SUPPLEMENTARY TABLES:

Supplementary table 1 – Eligibility criteria

Supplementary table 2 – Infection control guidelines

Supplementary table 3 – Study timetable for challenge volunteers

Supplementary table 4 – Study timetable for contact volunteers

For peer review only

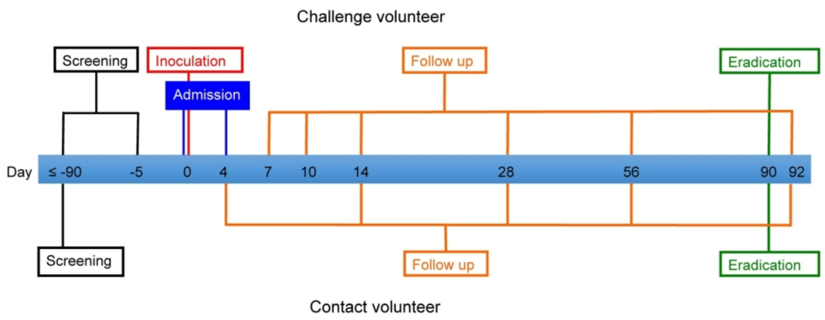

Study Timeline

190x107mm (300 x 300 DPI)

SUPPLEMENTARY TABLE 1: ELIGIBILITY CRITERIA

Inclusion criteria

Challenge volunteers	Contact volunteers
Healthy adults aged 18 to 45 years inclusive on the day of enrolment	Healthy adults aged 18 years or over on the day of enrolment
Fully conversant in the English language	
Able and willing (in the investigator's opinion) to comply with all study requirements	
Provide written informed consent to participate in the trial	
Provide written agreement to abide by infection control guidelines including agreement to abstain from intimate contact with any individual other than one declared and consented bedroom contact during the study period	
Provide written consent to allow the study team to discuss the volunteer's medical history with the General Practitioner	
Written informed contact volunteer consent provided by any bedroom contact	
Agreement to be admitted to Southampton NIHR-CRF for 4.5 days following inoculation	
For females only, willingness to practice continuous effective contraception (see below) during the study and a negative pregnancy test on the day(s) of screening and inoculation	For females only, willingness to practice continuous effective contraception (see below) during the study and a negative pregnancy test on the day of screening and challenge volunteer discharge
Agreement to take antibiotic eradication therapy according to the study protocol	
Able to correctly answer all questions in the pre-consent and infection control questionnaires	Able to correctly answer all questions in the infection control questionnaire
TOPS registration completed and no conflict found	

NIHR-CRF: National Health Institute for Health Research-Clinical Research Facility, TOPS: The Over-volunteering Prevention System

For peer review only

Effective contraception for female volunteers

Established use of oral, injected or implanted hormonal methods of contraception
Placement of an intrauterine device or intrauterine system
Total abdominal hysterectomy
Barrier methods of contraception (condom or occlusive cap with spermicide)
Male sterilisation if the vasectomised partner is the sole partner for the subject
True abstinence when this is in line with the preferred and usual lifestyle of the subject

For peer review only

Exclusion criteria

<table>
<tr><th>Challenge volunteers</th><th>Contact volunteers</th></tr>
<tr><td>Current active smokers defined as having smoked a cigarette or cigar in the last four weeks</td><td></td></tr>
<tr><td>N. lactamica or N. meningitidis detected on throat swab or nasal wash taken at screening or at the pre-challenge visit</td><td></td></tr>
<tr><td>Individuals who have a current infection at the time of inoculation</td><td></td></tr>
<tr><td colspan="2">Individuals who have been involved in other clinical trials involving receipt of an investigational product over the last 12 weeks or if there is planned use of an investigational product during the study period</td></tr>
<tr><td>Individuals who have previously been involved in clinical trials investigating meningococcal vaccines or experimental challenge with N. lactamica</td><td></td></tr>
<tr><td>Individuals who have received one or more doses of the meningococcus B vaccine Bexsero</td><td></td></tr>
<tr><td>Use of systemic antibiotics within the period 30 days prior to the challenge</td><td></td></tr>
<tr><td colspan="2">Any confirmed or suspected immunosuppressive or immune-deficient state, including HIV infection; malignancy, asplenia; recurrent, severe infections and chronic (more than 14 days) immunosuppressant medication within the past 6 months (topical steroids are allowed)</td></tr>
<tr><td>Use of immunoglobulins or blood products within 3 months prior to enrolment.</td><td></td></tr>
<tr><td>History of allergic disease or reactions likely to be exacerbated by any component of the inoculum</td><td></td></tr>
<tr><td colspan="2">Contraindications to the use of ciprofloxacin, specifically a history of epilepsy, prolonged QT interval, hypersensitivity to quinolones or a history of tendon disorders related to quinolone use</td></tr>
<tr><td colspan="2">Contraindications to the use of ceftriaxone, specifically hypersensitivity to any cephalosporins</td></tr>
<tr><td>Any clinically significant abnormal finding on clinical examination or screening investigations. In the event of abnormal test results, confirmatory repeat tests will be requested.</td><td></td></tr>
<tr><td colspan="2">Any other significant disease, disorder, or finding which may significantly increase the risk to the volunteer because of participation in the study, affect the ability of the volunteer to participate in the study or impair interpretation of the study data.</td></tr>
<tr><td colspan="2">Occupational, household or intimate contact with immunosuppressed persons, specifically HIV infection with a CD4 count <200 cells/mm3; asplenia; any malignancy, recurrent, severe infections and chronic (more than 14 days) immunosuppressant medication within the past 6 months (topical steroids are allowed)</td></tr>
<tr><td colspan="2">Occupational or household contact with children under 5 years or an older child with a tendency to co-sleep with the volunteer</td></tr>
<tr><td colspan="2">Pregnancy, lactation or intention to become pregnant during the study</td></tr>
<tr><td>Inability of the study team to contact the volunteer’s GP to confirm medical history and safety to participate</td><td></td></tr>
</table>

For peer review only

SUPPLEMENTARY TABLE 2: INFECTION CONTROL GUIDELINES

During admission – challenge volunteers only:

- The volunteer must wear a surgical mask covering the nose and mouth at all times unless within their personal room, while showering or having respiratory samples taken or while outside in open air
- The volunteers are not allowed to enter the personal rooms of other volunteers
- The volunteer must wash his/her hands before leaving their personal room
- The volunteer is not allowed to leave the NIHR-CRF without permission of the clinical team
- Volunteers are allowed to leave the NIHR-CRF for a maximum of two hours twice a day, between 08.00-18.00
- The volunteer will be escorted by a member of the study team when walking through non-designated areas of the NIHR-CRF
- The volunteer must not have contact with immunosuppressed individuals
- The volunteer must not have any direct contact that could involve transfer of respiratory secretions to anyone during the admission period
- The volunteer must not use the main entrance of the hospital or shops or cafes within the hospital building
- When outside of the NIHR-CRF the volunteer must be contactable by mobile phone at all times and must have study emergency phone number stored on their phone to contact the clinical study team if necessary
- The volunteer must be able to be return to the NIHR-CRF within 30 minutes.
- The volunteer may receive a maximum of two guests at a time between 8.00 and 22.00, who must wear masks covering nose and mouth while in close proximity to the volunteer and must adhere to strict infection control procedures.

Following discharge – challenge and contact volunteers:

For the first two weeks following discharge volunteers must avoid crowded social environments such as pubs and clubs.

For the remainder of the study period:

- Volunteers must not have any contact with high risk of transmission with any individuals other than their declared and consented bedroom contact/corresponding challenge volunteer – such contact includes:
    - Bed sharing
    - Intimate/sexual contact
    - Contact that may involve transfer of respiratory secretions e.g. kissing
    - Sharing cutlery or drinking vessels
- Volunteers must not engage in oral sex
- Volunteers must avoid contact with immunosuppressed individuals

For peer review only

SUPPLEMENTARY TABLE 3 – STUDY TIMETABLE FOR CHALLENGE VOLUNTEERS

	Screening	Pre challenge	Admission					Follow up							Potential additional visits		
Timeline (days)	≤ 90	-5	0	1	2	3	4	7	10	14	28	56	90	92	Additional shedding check[b]	Triggered eradication[c]	Post triggered eradication check
Day		W	M	Tu	W	Th	F	M	Th	M	M	M					
Visit window		+/-2	0	0	0	0	0	+/-1	+/-1	+/-2	+/-3	+/-5	+/-7	-1 to 0[a]	0[d]	0[d]	0[a]
TOPS confirmation	+																
Volunteer Information Sheet	+																
Informed consent	+																
Infection control training	+						+										
Vital signs	+	(+)	+	+	+	+	+	(+)	(+)	(+)	(+)	(+)	(+)	(+)	(+)	(+)	(+)
Medical history	+																
Physical examination	+	(+)	(+)	(+)	(+)	(+)	(+)	(+)	(+)	(+)	(+)	(+)	(+)	(+)	(+)	(+)	(+)
Pregnancy test (females	+		+										+			+	
Urinalysis	+																
Electrocardiogram	+																
Review eligibility		+	+														
Inoculation			+														
Eradication													+			+	
Review of adverse events and concomitant medications		+	+	+	+	+	+	+	+	+	+	+	+	+	+	+	+
Throat swab 1	+	+	+		+	+	+	+	+	+	+	+	+	+	+	+	+
Nasal wash		+				+				+	+	+	+			+	
Throat swab 2 (microbiome)			+			+		+	+	+	+	+	+			+	
Nasosorption test			+			+				+	+	+	+			+	
Saliva sample			+			+				+	+	+	+			+	
Environmental samples				+	+	+	+	+	+	+	+	+	+		+	+	
Safety bloods	8		8			8		8		8	8	8	8			8	
Immunological blood tests			70					70		70	70	70	70			70	
Cumulative blood volume	8		86			94		172		250	328	406	484				

(+) If clinically indicated, [a]1-2 days after eradication, [b]If increased shedding seen at one timepoint from Day 14, [c]If early eradication triggered (see section 9.5.3), [d]As soon as possible after triggering results are known.

For peer review only

SUPPLEMENTARY TABLE 4 – STUDY TIMETABLE FOR CONTACT VOLUNTEERS

	**Screening**	**Challenge volunteer discharge**	**Follow up**					**Potential additional visits**	
Timeline (days)	≤ 90	4	14	28	56	90	92	Early / triggered eradication[c]	Early / triggered eradication check
Day		F	M	M	M	+/-7[a]	-1 to 0[c]	0[d]	-1 to 0[b]
Visit window		0	+/-2	+/-3	+/-5				
TOPS confirmation	+								
Volunteer Information Sheet	+								
Informed consent	+								
Reconfirm eligibility		+							
Infection control training	+	+							
Vital signs	+		(+)	(+)	(+)	(+)	(+)	(+)	(+)
Medical history	+								
Physical examination	+		(+)	(+)	(+)	(+)	(+)	(+)	(+)
Pregnancy test (females only)	+	+				+		+	
Urinalysis	+								
Electrocardiogram	+								
Eradication						+		+	
Review of adverse events and concomitant medications			+	+	+	+	+	+	+
Throat swab	+	+	+	+	+	+	+	+	+

(+) If clinically indicated, [a]Same day as corresponding challenge volunteer, [b]1-2 days after eradication, [c]If early eradication triggered, [d]As soon as possible after triggering results are known

For peer review only
